# The influence of physical activity, sedentary behavior on health-related quality of life among the general population of children and adolescents: A systematic review

**DOI:** 10.1371/journal.pone.0187668

**Published:** 2017-11-09

**Authors:** Xiu Yun Wu, Li Hui Han, Jian Hua Zhang, Sheng Luo, Jin Wei Hu, Kui Sun

**Affiliations:** 1 School of Public Health and Management, Weifang Medical University, Weifang City, Shandong, China; 2 Affiliated Second Hospital of Weihai City, Faculty of Medicine, Qingdao University, Weihai City, Shandong, China; TNO, NETHERLANDS

## Abstract

**Background:**

The association between physical activity, sedentary behavior and health-related quality of life in children and adolescents has been mostly investigated in those young people with chronic disease conditions. No systematic review to date has synthesized the relationship between physical activity, sedentary behavior and health-related quality of life in the general healthy population of children and adolescents. The purpose of this study was to review systematically the existing literature that evaluated the relations between physical activity, sedentary behavior and health-related quality of life in the general population of children and adolescents.

**Methods:**

We conducted a computer search for English language literature from databases of MEDLINE, EMBASE, PSYCINFO and PubMed-related articles as well as the reference lists of existing literature between 1946 and the second week of January 2017 to retrieve eligible studies. We included the studies that assessed associations between physical activity and/or sedentary behavior and health-related quality of life among the general population of children and adolescents aged between 3–18 years. The study design included cross-sectional, longitudinal and health intervention studies. We excluded the studies that examined associations between physical activity, sedentary behavior and health-related quality of life among children and adolescents with specific chronic diseases, and other studies and reports including reviews, meta-analyses, study protocols, comments, letters, case reports and guidelines. We followed up the Preferred Reporting Items for Systematic reviews and Meta-Analyses (PRISMA) statement in the reporting of this review. The risk of bias of the primary studies was assessed by the Newcastle-Ottawa Scale. We synthesized the difference in health-related quality of life scores between different levels of physical activity and sedentary time.

**Results:**

In total, 31 studies met the inclusion criteria and were synthesized in the review. Most of the included studies used a cross-sectional design (n = 21). There were six longitudinal studies and three school-based physical activity intervention studies. One study used both cross-sectional and longitudinal designs. We found that higher levels of physical activity were associated with better health-related quality of life and increased time of sedentary behavior was linked to lower health-related quality of life among children and adolescents. A dose-response relation between physical activity, sedentary behavior and health-related quality of life was observed in several studies suggesting that the higher frequency of physical activity or the less time being sedentary, the better the health-related quality of life.

**Conclusions:**

The findings in this study suggest that school health programs promoting active lifestyles among children and adolescents may contribute to the improvement of health-related quality of life. Future research is needed to extend studies on longitudinal relationships between physical activity, sedentary behavior and health-related quality of life, and on effects of physical activity interventions on health-related quality of life among children and youth.

## Introduction

The associations between physical activity (PA), sedentary behavior (SB) and physical and mental health among children and adolescents have been well established. Systematic reviews and primary studies of PA and health have indicated that children and adolescents who engaged in increased levels of physical activities had better physical and mental health and psychosocial well-being than those in an inactive lifestyle [1–7]. Promoting PA among children and adolescents has been demonstrated to benefit a number of disease conditions, including obesity [8,9], coronary heart disease and other health problems [1,4]. Sedentary behavior characterized often as screen-based media use behaviors including watching television (TV), using computers/smartphones and playing video games [5] are associated with various negative health consequences [2,5–7,10]. The adverse consequences resulted from sedentary behaviors include an increased risk of obesity, cardiovascular disease and all-cause mortality, and a range of impaired psychological health [2,3,5–7,10,11]. Sedentary behavior also contributes to a delay of cognitive development and a decrease in academic achievement of children and youth [12].

Health-related quality of life (HRQOL) has been increasingly used as a health outcome among children and adolescents to assess their physical and social functioning, mental health and well-being, and to evaluate population-based intervention programs [13]. HRQOL is a multidimensional construct that covers physical, psychological, and social health and hence represents overall health of an individual [14]. Assessment of HRQOL among children and adolescents is important in identifying subgroups with poor health status and in guiding effective intervention strategies to improving health of the younger population. The association between PA and HRQOL in children and adolescents has been mainly investigated among those with chronic disease conditions such as obesity, asthma and cancer [15–18]. These studies have reported that children and adolescents who undertake an active lifestyle experience better HRQOL than their peers who engage in an inactive lifestyle. In the general population, the relationship between PA and HRQOL has been well investigated in adults [19] relative to children and youth (e.g.,school or population-based samples). Moreover, much less is known about the relationship between sedentary behavior and HRQOL [20]. In the past decade, we have found accumulating studies that examined the effect of PA and sedentary behavior on HRQOL among populations of children and adolescents. To our best knowledge, no systematic review to date has been published to evaluate the relationship between PA, sedentary behavior and HRQOL among the general popluation of relatively healthy children and adolescents. Particularly, it is essential to explore how PA and sedentary behavior influence different aspects of physical, psychological and social functioning of HRQOL among children and adolescents, and whether a dose-response relation exists between PA levels, time spent on sedentary behaviors and HRQOL. Such information will help to provide an evidence-base for public health policy to invest in school-based health promotion programs in order to enhance health and quality of life among children and adolescents.

The purpose of the present study was to 1) review and synthesize the existing literature that investigated associations between PA, sedentary behavior and HRQOL in the general population of children and adolescents; 2) provide evidence-based recommendations for guiding school-based health behavior intervention programs to enhance HRQOL among children and adolescents.

## Study methods

### Literature search

A computer search was performed for English language literature using databases of MEDLINE (1946-Week 2, January, 2017), EMBASE (1974-Week 2, January, 2017), PSYCINFO (1987-Week 2, January, 2017). MeSH headings and keywords used included ‘physical activity’, ‘exercise’, ‘sedentary behavior’, ‘screen time’, ‘television’, ‘computers’, ‘video games’, ‘lifestyle’, ‘health-related quality of life’, ‘quality of life’, ‘health status’,‘children’, ‘adolescents’. From the relevant articles, we searched the PubMed related articles and manually examined the reference lists of the existing literature to retrieve other eligible studies.

The electronic search was conducted by a single researcher (XYW). The reviewer screened the citations and abstracts and selected the eligible articles based on the inclusion and exclusion criterion. In case there were studies that the primary reviewer was uncertain whether a paper was eligible for the review, the full text articles were obtained. The full-text articles of all potentially eligible studies were retrieved, and then reviewed by the two reviewers (XYW, LHH) separately for inclusion criteria. Disagreements regarding the eligibility of the studies for inclusion were resolved by discussion among all the researchers.

### Inclusion and exclusion criteria

In this review, we aimed to collect studies that focused on examinations of the associations between physical activity, sedentary behavior and HRQOL among the population of healthy children and adolescents. The inclusion criteria were as follows: (1) The outcome of interest: Studies used one or more multi-dimensional HRQOL measures, and the outome has to be indicated as quality of life (QOL) or health-related quality of life. (2) Population of interest: The genereal population of children and adolescents aged between 3–18 years, including school-aged, or community-based children and adolescents. The general population of children and adolescents refers to all children and adolescents in communities or schools in a geographic region or a country who are relatively healthy in comparison with those children and adolescents with specific diseases (e.g., patients with diabetes, obesity or cerebral palsy, etc.). For longitudinal and intervention studies with a follow-up age of greater than 18 years, the age of children and youth had to be 3 to 18 years at baseline when at least one exposure of PA and sedentary behavior was measured. (3) Types of studies: Cross-sectional, longitudinal and health intervention studies. The intervention studies for quality of life were those studies that targeted to promoting PA and reducing sedentary behavior among children and adolescents. (4) Measure of the exposure: PA is defined as any bodily movement worked with muscles that requires energy expenditure [21]. Sedentary behavior is any waking behavior while sitting, lying, reclining or standing with low energy expenditure [22,23]. Both subjective and objective measures of PA and sedentary behavior were included.

The exclusion criteria were: (1) Studies that examined associations between PA, sedentary behavior and HRQOL among children and adolescents with specific chronic disease conditions (e.g.,diabetes, asthma, obesity). (2) Studies that assessed associations between PA, sedentary behavior and HRQOL among adults. (3) Studies that used only a single item of self-rated/self-perceived health as a marker of HRQOL. (4) Studies that used combined indicators of PA, sedentary behavior and other variables (e.g.,diet factors,sleep) that were generated from cluster analysis. (5) Reviews, meta-analyses, study protocols, comments, letters, case reports and guidelines.

### Data extraction

A single researcher (XYW) abstracted data from all eligible full text articles that included study publication year, primary author, country, study design, sample, PA and sedentary behavior assessments, HRQOL measures, statistical methods and main findings. Another reviewer (LHH) checked the abstracted information for completion and accuracy.

### Risk of bias assessment

We used the Newcastle-Ottawa Scale to assess the study quality [24]. This scale is a checklist with eight items that consists of three quality components: selection, comparability and outcome. Each item can be scored as one or two points and summed up to a total score, ranging from 0 to 9, with a higher score indicating low risk of bias or better quality [24]. Previous research categorized the risk of bias score of individual studies into high, moderate and low risk of bias [25]. We grouped the summed scores into high (0–4), moderate (5–6), and low (7–9) risk of bias.

### Data synthesis

The main findings were synthesized using descriptive tables for qualitative comparisons. The study characteristics, the assessment of the exposure and the outcome, the association between PA, sedentary behavior and HRQOL, and the risk of bias were presented for each included study in the evidence table. For studies that utilized similar measures of the exposure (e.g., PA and screen time) and the outomes (e.g., the PedsQL 4.0 total score), we performed meta-analyses for the overall association between PA, screen time and HRQOL. Pooled estimates were obtained for the differences in total HRQOL scores and their 95% confidence intervals between different levels of physical activity and sedentary time. A forest plot was generated for the overall effect across the studies. We used the random-effects model in meta-analyses to account for heterogeneity across the studies. The tau-square (Tau^2^) value and I^2^ (squared) index was used to test the degree of heterogeneity [26]. Review Manager Software 5.2 (The Cochrane Collaboration, Copenhagen Denmark) was used for the meta-analysis. Reporting of this review was guided by the Preferred Reporting Items for Systematic reviews and Meta-Analyses (PRISMA) statement [27].

## Results

### Characteristics of the included primary studies

**Fig 1:**
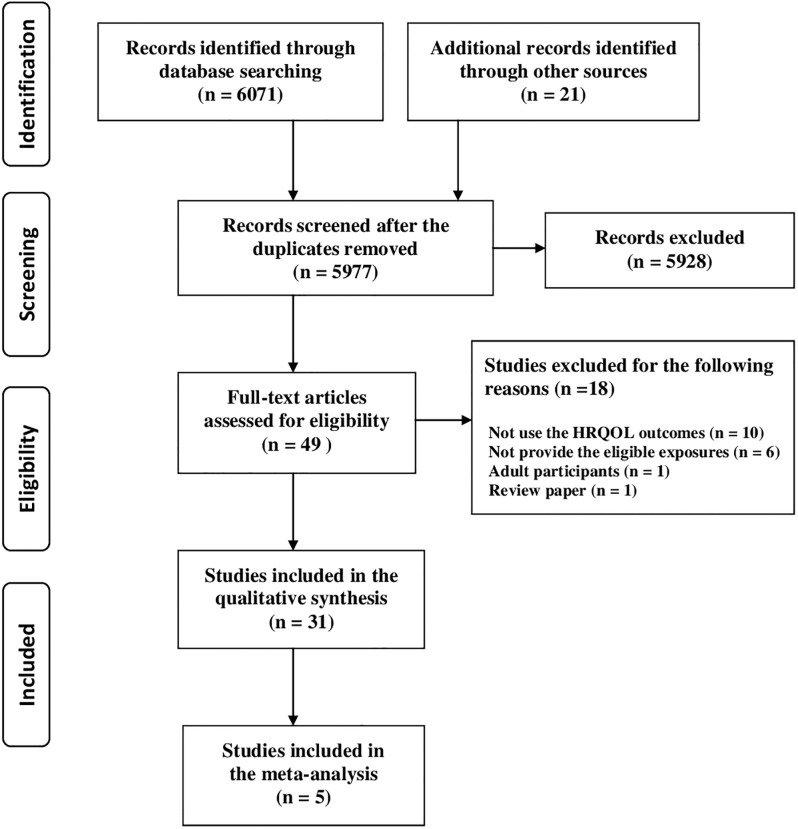
PRISMA flow diagram for selection and inclusion of the eligible studies.

A total of 6,071 relevant citations (MEDLINE: 3,108, EMBASE: 2,545, PSYCINFO: 418) were identified through electronic database searching and screened for eligibility. Additional 22 articles were identified through searching PubMed-related articles and reference lists from existing relevant studies. After the title and abstract review, and removing duplicate references, 49 full text articles were retrieved for detailed examinations. Among these studies, 18 were excluded due to inappropriate outcomes (e.g., anxiety,self-esteem), inappropriate exposure (e.g., cluster of PA,screen-based behavior and diets), adult participants or a review [28–45]. In total, 31 studies were included in the final synthesis [46–76]. Selection process of the studies was displayed in the PRISMA flow diagram (Fig 1).

Table 1 shows the characteristics of the included studies. In total, 79,046 participants were included in the statistical analysis in the primary studies with a sampe size ranging from 152 (Dalton et al., 2010) to 16,560 (Galán et al., 2013) students. The included studies came from 15 countries. Seven studies were conducted in Australia and four studies were from Spain. The remaining 20 studies were conducted in the following countries: United States (n = 3), Japan (n = 3), Germany (n = 2), Switzerland(n = 2), Canada (n = 2), UK (n = 1), Brazil (n = 1), Italy (n = 1), France (n = 1), Norway (n = 1), China (n = 1), Malaysia (n = 1), Iran (n = 1).

**Table 1:** Characteristics of the included studies, assessment of physical activity and sedentary behavior among children and adolescents.

First author, publication year and country	Study design	Sample size, age, gender	PA assessment or interventions	Sedentary behavior assessment
Muros et al., 2017 Spain [46]	Cross-sectional	Adolescents n = 456 Age range:11–14 years 51.5% girls	Self-report The Physical Activity Questionnaire for Older Children (PAQ-C): 9 items rated on a five-point scale. A summary score was calculated from the mean of the 9 items, with a score of five indicating high physical activity (PA).	Self-report Sedentary behavior (SB) was assessed as the number of hours per day that adolescents spent watching TV/DVDs, computer screens, smart phones, tablets, or other devices.
Jalali-Farahani et al., 2016 Iran [47]	Cross-sectional	Adolescents n = 465 Age range:14–17 years 48.8% girls	Self-report The Quantification de l’Activite Physique en Altitude Chez les Enfants (QAPACE) questionnaire:18 items covering all possible school and vacation activities (hours/week)	Self-report Screen time viewing, watching TV, videogames/internet (hours/week)
Omorou et al., 2016 France [48]	Longitudinal 2-year follow up	Adolescents n = 1,445 Age range:14–18 years at baseline 56% girls	Self-report The French version of the International Physical Activity Questionnaire (IPAQ), measuring the duration of PA (minutes/day) for three types of activities: vigorous, moderate and walking. French recommendations: at least 1 h of moderate to vigorous physical activity (MVPA) per day. WHO recommendations: 1 h of MVPA per day and in vigorous activity at least 3 times per week. Cumulative PA was defined by the number of follow-up times (baseline and time 2) that adolescents achieved the PA recommendations, and categorised as: none, once, twice.	Self-report High SB was defined as ≥7 h/day sitting time. Cumulative level of SB was defined by the number of follow-up times (baseline and time 2) that adolescents reported having ≥7 h/day of sitting time.
Wafa et al., 2016 Malaysia [49]	Cross-sectional	Children n = 156 Convenience sampling from 5 primary schools: 78 obese children matched with 78 normal weight children. Age range:9–11 years Mean age = 9.9 (SD 0.5)	PA was measured by accelerometer for five days. Cut-points for PA: Sedentary: <1100 cpm (counts per minute), Light intensity PA:1100–3200 cpm, Moderate to vigorous intensity PA: >3200 cpm.	SB was measured by accelerometer for five days. SB was defined as a cut-point less than 1100 cpm from accelerometry data.
Sigvartsen et al., 2016 Norway [50]	Cross-sectional	First-year high school students n = 156 Age range:15–18 years Mean age = 16 (SD 0.8) 79% girls	Self-report PA was measured by the number of times of a week and the number of hours in the leisure time that the adolescents were physically active (defined as out of breath or sweating)	Self-report SB was measured by screen time that was calculated as the number of hours per day
Casey et al., 2014 Australia [51]	Clustered-RCT 12-month intervention	Adolescent girls 362 intervention students: Mean age = 13.4 (SD 0.9) 259 control students: Mean age = 13.4 (SD 0.9)	A school-community based program during a 12-month period. 16 randomly chosen schools (8 in the intervention group, 8 in the control group). The schools in the intervention received a school physical education (PE) component, involving in student-centred teaching approaches and behavioral skill development. The schools in the control group went with the usual curricular programming and did not include any engagement strategy.	NA
Chen G et al., 2014 Australia [52]	Cross-sectional	Children and adolescents n = 3,357 51% girls Primary school students: Mean age = 10.6 (7.9–13.0) High school students: Mean age = 15.1 (13.6–16.9)	Self-report PA was defined as number of days that participants were physically active for a minimum of 60 minutes per day over the last 7 days	Self-report Sedentary days was defined as number of days that participants experienced two or more hours of screen time (TV, computer, video games) per day outside school hours over the last 7 days, and average screen hours across school and non-school days
Gopinath et al., 2014 Australia [53]	Longitudinal 5-year follow-up	Adolescents n = 742 12-year old children at baseline, 17–18 years at follow up	Self-report MVPA was estimated as the average hours/day. Low PA was defined as <60 minutes of total PA a day. Each of the five obesogenic behavioral risk factors (i.e. low fruit and vegetable intake, low physical activity, high screen time, high soft drink intake and high snack intake) was coded ‘1’ for present and ‘0’ for absent.	Self-report Screen time was measured as the number of hours of daily watching TV, playing video games, and using computers. High screen time was defined as ≥2 hours per day.
Vella et al., 2014 Australia [54]	Longitudinal 2-year follow up	Children n = 4,042 Age range:8–9 years in 2008 (baseline) Mean age = 8.3 (SD 0.4) years at baseline	Parent-report Sports participation was measured by two items assessing regular participation in team and individual sports. Parents were asked ‘In the last 12 months, has (your) child regularly participated in team sport?’; ‘In the last 12 months, has (your) child regularly participated in individual sport? Children were defined as participating in sports if parents answered ‘yes’ to at least one of the items.	NA
Xu et al., 2014 China [55]	Cross-sectional	Primary and high school students (grades 4–12) n = 839 Age range:9–17 years	Self-report PA was recorded by the frequency and duration for each of 24 listed activity items per day for 7 days. Each activity was conveyed into a specific metabolic equivalent (MET) value based on the updated compendium of physical activities. The overall PA was the sum of MET values of all types of activities, and was grouped into high, medium, and lower levels corresponding to the top 25%, middle 50% or the low 25% of the MET distribution of the participants.	Self-report SB was measured as the average number of hours spent doing homework per day in the past 7 days
Gu et al., 2014 United States [56]	Cross-sectional	Adolescents n = 282 Age range:11–15 years Mean age = 12.4 (SD1.0) 50.7% girls	Self-report The Physical Activity Questionnaire for Older Children (PAQ-C), assessing adolescents’ MVPA such as recreational activities, sports, and other types of exercise in the previous week	NA
Finne et al., 2013 Germany [57]	Cross-sectional	Children and adolescents n = 6,813 Age range:11–17 years Mean age = 14.62 (SE 0.02) for girls, boys and total 48.7% girls	Self-report PA was measured as the number of times a week that the participants were physically active enough in their leisure time that they sweated or breathed hard. The responses were: ‘about every day’, ‘about 3–5 times a week’, ‘about once or twice a week’, ‘about once or twice a month’, or ‘never’.	Self-report Screen-based media use (SBM) was assessed as the average amount of daily time (hours) spent on TV/videos, computer/internet and gaming. The total screen time index was computed for adolescents with valid answers for all three media. SBM was classified into four groups: <2 h/day, 2-<3 h/day, 3-<4.5 h/day, ≥4.5 h/day.
Galán et al., 2013 Spain [58]	Cross-sectional	Children and adolescents n = 16,560 Age range:11–18 years 46.1% girls	Self-report The MVPA was measured as the number of days that participants engaged in moderate and vigorous activity for at least 60 minutes per day in the last seven days	NA
Petracci et al., 2013 Italy [59]	Cross-sectional	School-aged children. n = 4,338 Age range:13 years Mean age = 13.8 years (SD 0.4) 49.7% girls	Self-report PA was measured by hours per week	NA
Spengler et al., 2013 Germany [60]	Cross-sectional	Adolescents. n = 1,828 Age range:11–17 years Mean age = 14.2 (SD 1.9) 48.7% girls	Self-report The PA questionnaire included questions on the frequency (how many times per week) and duration (in minutes) of adolescents’ PA in sports clubs and during their leisure time outside of sports clubs	NA
Gopinath et al., 2012 Australia [61]	Cross-sectional Longitudinal, 5-year follow up	Adolescents **Cross-sectional sample:** nc = 1,094 Age range:17–18 years Mean age = 17.3 (SD 0.6) 56.1% girls **Cohort sample**: nl = 775 Age range:17–18 years at follow-up	Self-report Time (hours) spent on various physical activities (indoor and outdoor) in an average week was collected. Average hours per day were calculated separately for total, indoor and outdoor activities.	Self-report Adolescents reported the number of hours usually spent daily in watching television, playing video games, using a computer, reading for pleasure, and doing homework. Total screen time was summated.
Wu et al., 2012 Canada [62]	Cross-sectional	Children in grade five n = 3,421 Age range:10–11 years	Combination of child’s self-report and parent-report of PA in the last 7 days. The Physical Activity Questionnaire for Children (PAQ-C) was used. A composite score ranging from 0 to 5 was derived from 29 items. Children with a composite score of 3 and greater were defined as physically active.	NA
Perry et al., 2012 United States [63]	Cross-sectional	Children and Adolescents n = 371 Age range:3–17 years Median age = 9.8 48% girls	Self-report For those children younger than 11 years, interviews were conducted with the assistance of a parent/guardian (proxy). Participants were asked ‘How often do you exercise, work or do other activities enough to work up a sweat?’ Children/youth with ‘daily’ or ‘5–6 times per week’ to the question were categorized as physically active. The moderately active (1–4 days/week) and minimally active (<1 day/week) groups were combined together to one group (inactive group) for statistical analyses.	Self-report For those children younger than 11 years, interviews were conducted with the assistance of a parent/guardian (proxy). Children/youth were asked ‘How many hours did you sit and watch television or videos, play video games, or use the computer yesterday?’ Children/youth were categorized as having high screen time if they reported >2 hours/day.
Lacy et al., 2011 Australia [64]	Cross-sectional	Adolescents n = 3,040 Age range:11–18 years 44% girls	Self-report Three questions asking PA during school recess, lunch time and after school over five school days. Adolescents who ‘mostly played active games’ during at least one school break (recess or lunch time) or who participated in PA after school on all five school days were considered physically active on every school day. Those who ‘mostly just sat down’ or stood or walked around’ at recess and lunchtime and participated in PA after school on four or fewer days were classified as not physically active on every school day.	Self-report Eight questions were used to measure leisure time screen-based behaviors. Averages of the number of hours spent daily on watching TV, playing video games and using computer (not for homework) were calculated.
Borras PA, et al., 2011 Spain [65]	Cross-sectional	Children n = 302 Age range:11–12 years 50% girls	Self-report The School Health Action, Planning and Evaluation System (SHAPES) physical activity questionnaire has 45 multiple choice questions, asking about moderate to vigorous PAs over 7 days	The SHAPES questionnaire includes questions on sedentary activities (Watching TV, playing video games, homework)
Dalton et al., 2010 United States [66]	Cross-sectional	Children in grade six n = 152 Age range:11–12 years 54% girls	Self-report Physically Active Days per Week (two questions): Over the past 7 days, on how many days were you physically active for at least 60 min per day? Over a typical or usual week, on how many days were you physically active for a total of at least 60 min per day?	Self-report Hours of screen time per day (two questions): On an average school day, how many hours do you watch TV? On an average school day, how many hours do you play video or computer games or use a computer for something that is not school work?
Hartmann et al., 2010 Switzerland [67]	Clustered-RCT 9-month intervention	Children (first and fifth grade) 540 children participated in the study, 456 (84%) had QOL baseline data, 411 had valid baseline and post-intervention data. **First grade students:** n1 = 111(50% girls) in the intervention group (mean age = 6.9, SD = 0.3), n2 = 69 (54% girls) in the control group (mean age = 6.9, SD = 0.3). Age range:6–8 years. **Fifth grade students:** n1 = 146 (57% girls) in the intervention group (mean age = 11.0, SD = 0.5), n2 = 85 (48% girls) in the control group (mean age = 11.3, SD = 0.6). Age range:10–12 years.	PA interventions: Physical education classes, PA homework, and encourage activities during school breaks	NA
Kriemler et al., 2010 Switzerland [68]	Clustered-RCT 9-month intervention	Children (first and fifth grade) 28 classes from 15 elementary schools were randomly assigned to an intervention (n = 16 classes) or control arm (n = 12 classes). 297 children in the intervention group, 205 in the control group. 498 children had complete baseline and follow up data. **Mean age for first grade:** 6.9 (SD: 0.3) years for both intervention and control groups at baseline. **Mean age for fifth grade:** 11.0 (SD: 0.5) years in the intervention, 11.3(0.6) years in the control group at baseline.	PA interventions: Multi-component PA programme including three physical education lessons each week and two additional lessons a week, daily short activity breaks, and PA homework	NA
Boyle et al., 2010 UK [69]	Cross-sectional	Children n = 1,771 Age range:11–15 years Mean age = 13.2 (SD 1.2) 48.3% girls	Self-report PA was assessed by the Western Australian Child and Adolescent Physical Activity and Nutrition Survey (CAPANS). The CAPANS consists of 24 questions recording the type of PAs in a typical week and the number of times spent on that activity. PA was grouped into two categories: ‘yes’ or ‘no’ based on the UK government PA recommendation for children (60 minutes of MVPA per day).	NA
Gordia et al., 2010 Brazil [70]	Cross-sectional	Adolescents Convenience sample n = 608 Age range:14–20 years	Self-report PA was measured using the International Physical Activity Questionnaire (IPAQ), version 8, short last seven days format, validated for Brazilian adolescents. PA was categorized to quartiles.	NA
Herman et al., 2010 Canada [71]	Longitudinal, 22-year follow up	Children and adolescents n = 310 Age range:7–18 years at baseline Mean age at baseline:13.0 (SD 3.4) for males, 12.6 (SD 3.5) for females. 50.6% females	Self-report Leisure time PA was measured using the Minnesota Leisure Time Physical Activity Questionnaire. PA was categorized into two groups: ≥ 3.0 kcal/kg/day, <3.0 kcal/kg/day.	NA
Mathers et al., 2009 Australia [72]	Cross-sectional	Adolescents n = 925 Mean age = 16.1 (SD 1.2) 49.6% girls	NA	Self-report The Multimedia Activity Recall for Children and Adolescents (MARCA). Electronic media use was measured as minutes per day, including TV, video game, computer and telephone.
Sánchez-López et al., 2009 Spain [73]	Cross-sectional	Children n = 1,073 (76.2% response rate, grade five and six). Age range:11–13 years. Mean age for boys = 11.08 (SD 0.78). Mean age for girls = 10.95 (SD 0.73). 50% girls.	Self-report Two items: In the past 4 weeks, how often did you play active games or sports? In the past 4 weeks, how often did you run hard to play or do sports? A higher score on these items indicates a higher level of physical activity.	NA
Wang et al., 2008 Japan [74]	Longitudinal 10-year follow up	9,674 families with children of 3 years old participated in the survey (Phase I) between 1992 and 1994. 9,718 students (response rate: 93.0%) in the first grade of junior high schools responded to the survey (Phase IV) in 2002. Complete data for both phases’ surveys were available for 7,289 children (3,686 boys and 3,603 girls). Age nearly 13.0 years at follow up. 49.4% girls.	Parent-report PA was estimated by parents of children in comparison with children’s peers. PA data were collected in Phase I survey. PA was categorized as three levels: active, average, less active.	NA
Chen X et al., 2005a Japan [75]	Longitudinal 3-year follow up	Children n = 7,794 with complete baseline (1999) and follow up (2002) data (follow-up rate: 74.7%). Mean age = 12.8 years at follow up. 50.4% girls.	Self-report Frequency of PA: very often, often, seldom, almost never	Self-report Television viewing time was categorized on three levels: <2 h, 2–3 h, >3 h per school day. Video game playing time on four levels: <1 h, 1–2 h, 2–3 h, >3 h per school day.
Chen X et al., 2005b Japan [76]	Cross-sectional	Children n = 7,887 in the analysis Mean age = 12.75 (SD: 0.29) 50.5% girls	Self-report Frequency of PA was measured on a four-point scale: very often, fairly often, not often, nearly not	Self-report Television viewing includes four categories: 0–2 hours, 2–3 hours, 3–4 hours, and >4 hours per day

**Abbreviations:** PA: physical activity; SB: sedentary behavior; MVPA: moderate to vigorous PA; RCT: randomised controlled trial; SD: standard deviation; h: hours; UK: United Kingdom; NA: not applicable.

Most of these studies were cross-sectional studies (n = 21). Six were longitudinal studies [48,53,54,71,74,75], and three studies evaluated the effect of school-based PA programs on HRQOL in school children and adolescents using clustered-randomized controlled trial (RCT) design [51,67,68]. One additional study examined both cross-sectional and longitudinal associations between physical activity, sedentary behavior and HRQOL among adolescents [61].

Of the included studies, 16 studies assessed the association between both physical activity and sedentary behavior and HRQOL among children and youth. A total of 13 studies examined the association between physical activity only and HRQOL among children and youth [51,54,56,58–60,62,67–70,73,74]. One study analyzed the long-term effect of youth physical activity (measured at age of 7–18 years) on their HRQOL 22 years later in adulthood [71]. One study investigated the association between electronic media use (television, video game, computer and telephone) and HRQOL among adolescents [72].

### Assessment of physical activity and sedentary behavior

Most included studies used self-report of physical activity (Table 1). Two observational studies used parent-report of physical activity for children [54,74]. One Canadian study used a composite measure of PA that was based on the combination of child’s self-report and parent-report questions [62]. One study collected PA data objectively using accelerometers [49]. Physical activity by self- or parent-report was measured as hours/minutes a day, or number of times a week or number of days over a week or a month (Table 1). In two Japanese studies, frequency of PA was measured on a four-point scale: very often, often, seldom, almost never [75,76].

Sedentary behavior was measured in most studies by number of hours or minutes spent daily in watching television, playing video games, using computers and telephones. Average time spent daily on sedentary activities was calculated in most of the studies. Some studies also calculated number of hours spent daily for television viewing, playing video games or using computers, respectively. One study assessed sedentary time by accelerometer [49].

### Measurement of health-related quality of life

With respect to the HRQOL instrument, the Paediatric Quality of Life Inventory 4.0 Generic Core Scales (PedsQL 4.0) was used in 12 studies (Table 2). Three studies applied the EQ-5D-Y, and three studies utilized the KIDSCREEN-10. Other HRQOL measures included the Japanese COOP charts (n = 3), the Child Health Questionnaire (CHQ-PF50) (n = 2), the Child Health Utility 9D (CHU9D) (n = 2), the Child Health and Illness Profile-Child Edition (CHIP-CE) (n = 2), German KINDL-R (n = 2), KIDSCREEN-27 (n = 1), the Duke Health Profile (DHP) (n = 1), the Medical Outcomes Study Short Form 36 (SF-36) (n = 1), and the WHOQOL-Bref (n = 1) (Table 2). Most of the HRQOL outcomes were reported as continuous variables (e.g., PedsQL total and subscale scores, KIDSCREEN-10 index, CHU9D). Some studies also used subscales of the HRQOL measures (e.g., the EQ-5D-Y dimensions) as categorical outcome variables in the analysis [62,74–76].

**Table 2:** Measurement of HRQOL and the main findings of the included studies.

First author and publication year	HRQOL measures	Outcome and analytical method	Main findings		Risk of bias (Score)
			PA and HRQOL	SB and HRQOL	
Muros et al., 2017 [46]	Self-report KIDSCREEN-27, 27 items, five components: physical well-being, psychological well-being, autonomy and parent’s relation, social support and peers, and school environment. The total score was transformed to T-score with mean scores of 50±SD 10. Higher scores indicate higher HRQOL.	**Outcome:** KIDSCREEN-27 total score **Method:** Hierarchical linear regression **Software used:** SPSS 22.0	A higher level of PA was associated with higher HRQOL scores	Not report	5
Jalali-Farahani et al., 2016 [47]	Self-report, parent-proxy report The Pediatric Quality of Life Inventory (PedsQL) version 4.0, PedsQL 4.0. 23 items, four subscales: physical, emotional, social, and school functioning. The five-point scale scores were transformed to 0–100,with higher scores indicating better HRQOL.	**Outcome:** Total and subscale scores **Method:** Pearson correlation **Software used:** SPSS 19.0	Spending more time on sport activities correlated to better HRQOL scores in both boys and girls	During school periods, spending more time on playing video game/surfing internet was related to poor school functioning in boys. During vacation periods, spending more time on watching TV, listening to music/reading were related to poorer QOL in boys.	4
Omorou et al., 2016 [48]	Self-report The Duke Health Profile (DHP), 17-item, 10 dimensions. 4 dimensions were analyzed: physical, mental, social and general health. The score was transformed to Z-score, with a Z-score≥0 indicating good HRQOL.	**Outcome:** DHP dimension scores **Method:** Bivariate and multivariate linear regression **Software used:** SAS 9.3	The cumulative level of high PA was associated with high HRQOL at 2-year follow up in all four dimensions: physical, mental, social and general health	Cumulative high SB was associated with reduced physical, mental and general dimensions of HRQOL at 2-years	7
Wafa et al., 2016 [49]	Self-report The PedsQL 4.0, 3 summary scores: total score, physical health summary, psychosocial health summary	**Outcome:** Total and subscale scores **Method:** Multiple linear regression **Software used:** SPSS 20.0	Less time in moderate to vigorous PA was associated with lower psychosocial health and lower PedsQL total score in the unadjusted regression model but not in the adjusted model	More time in SB was associated with lower psychosocial health and PedsQL total score in the unadjusted regression model but not the adjusted model	4
Sigvartsen et al, 2016 [50]	Self-report The Norwegian version of the KIDSCREEN-10, 10 questions covering physical, emotional, mental, social and behavioral components of QOL in the last week on a five-point scale. A HRQOL index was computed ranging from 0 to 100, with higher value representing better HRQOL.	**Outcome:** Kidscreen-10 index **Method:** Multiple linear regression with backward elimination method **Software used:** SPSS 21.0	Students who reported enjoying physical education experienced higher HRQOL	The association between screen time and HRQOL was not statistically significant	4
Casey et al., 2014 [51]	Self-report The PedsQL 4.0	**Outcome:** Total and subscale scores **Method:** Linear mixed model with adjustment for baseline value of the measure **Software used:** SPSS 18.0	The intervention group had significantly higher scores on three PedsQL scores: physical functioning (adjusted Mean±SE: 83.9 ± 0.7, p = 0.005), psychosocial (79.9 ± 0.8, p = 0.001) and total score (81.3 ± 0.7, p = 0.001) than the control group (80.9 ± 0.8; 76.1 ± 0.9 and 77.8 ± 0.8 respectively), suggesting that the intervention program positively influenced quality of life.	NA	8
Chen G et al., 2014 [52]	Self-report Child Health Utility 9D, CHU9D, 9 dimensions: Worried, Sad, Pain, Tired, Annoyed, School work, Sleep, Daily routine, Ability to join in activities. 5 different levels in each question indicating severity.	**Outcome:** CHU9D utility score **Method:** Multiple linear regression random effect model **Software used:** Stata 12.1	Each additional day of being physically active was associated with an additional mean utility of 0.004 (p<0.001 and p = 0.05) for primary and high school samples	An additional day of having ≥2 hours of screen time was associated with a decrease of utility score of 0.005 and 0.008 (p<0.001 both) for primary and high school students	6
Gopinath et al., 2014 [53]	Self-report The PedsQL 4.0	**Outcome:** Total and subscale scores **Method:** General linear regression model **Software used:** SAS 9.2	Children with a combination of five lifestyle risk factors compared with zero lifestyle risk factor at baseline had a lower physical summary score (ptrend = 0.001) five years later. In boys, there was a significant reduction in both total and physical summary score with multiple unhealthy behaviors of 4 or 5 risk factors, 4.5-units (ptrend = 0.02) and 4.2-units (ptrend = 0.01) for total and physical summary score, respectively. Girls with 4 or 5 versus 0 or 1 lifestyle risk factors at baseline, reported 4.6-units lower PedsQL physical summary score at the 5-year follow-up.	Same as the result for PA	6
Vella,et al., 2014 [54]	Parent-report The PedsQL 4.0, parent-version	**Outcome:** Total and subscale scores **Method:** Multivariate general linear regression **Software used:** SPSS 19.0	A significant association was observed between participation in sports and HRQOL. Children who maintained participation in sports throughout the 2-year follow up reported better HRQOL at follow-up than children who did not participate in sports, dropped out of sports, and commenced participation after the baseline. The magnitude of total HRQOL differences between sport participants and nonparticipants at age 10 years was approximately 5 units, greater than the minimum clinically meaningful difference of 4.5 units on PedsQL.	NA	8
Xu et al., 2014 [55]	Self-report The Child Health Utility 9D (CHU9D)	**Outcome:** CHU9D utility **Method:** Multivariable linear regression, two-level random effect model. **Software used:** Stata 12.1	Students with a high level of PA had a higher mean utility (0.023 or 0.029) for standard gambling (SG) and best worst scaling (BWS) scoring algorithms (P<0.05)	Each additional hour of doing homework was associated with a decrease of 0.019 and 0.021 points in mean utility that was based on SG and BWS scoring algorithms respectively (both P<0.01)	5
Gu et al., 2014 [56]	Self-report The PedsQL 4.0	**Outcome:** Subscale scores **Method:** Pearson correlation, Multiple regression **Software used:** not reported	PA was positively associated with physical, emotional, social functions of HRQOL (Correlation coefficient ranged from 0.15 to 0.18, p<0.01), but not school function. Multiple regression analysis did not show a significant association between PA and HRQOL.	NA	3
Finne et al., 2013 [57]	Self-report German KINDL-R, 6 dimensions: physical, emotional well-being, self-esteem, family, friends and school. Each dimension has four items. The score ranges between 0 (lowest HRQOL) to 100 (highest HRQOL).	**Outcome:** Total and subscale scores **Method:** Multilevel regression models estimated separately for boys and girls. **Software used:** Stata/IC 12.1	In both genders, HRQOL was related to PA in a dose-response manner for most subscales, with significant linear trends. Higher frequency of PA was related to higher HRQOL with small to moderate effect sizes for daily PA relative to no regular PA in both genders. The effects were larger in boys than in girls, with the exception of the school domain.	Negative associations of SBM with HRQOL were significant for all HRQOL domains in girls. A dose-response relation (linear trend) was observed for most subscales except for the family among girls. In boys, a dose–response relation was only seen with physical well-being and school domains. For all HRQOL subscales, SBM effects were larger in girls than in boys.	7
Galán et al., 2013 [58]	Self-report The KIDSCREEN-10, 10 questions including mood, ability to concentrate, energy, vitality, well-being and ability to have fun with friends. Each question has five levels of responses ranging from ‘never’ to ‘always’ or from ‘not at all’ to ‘extremely often’. The scores were transformed to T-values with scale means of 50 and SD of 10, higher values indicating higher HRQOL.	**Outcome:** KIDSCREEN-10 index **Method:** Linear regression model adjusting for a set of personal and familial risk factors. Linear and quadratic trends of the association between MVPA and HRQOL were calculated **Software used:** Stata 11.0	In both genders, an increasing dose-response association between MVPA and the HRQOL was observed. Mean Kidscreen-10 index score for each category of the MVPA: Never: 42.6, 1–2 days: 44.3, 3–4 days: 46.2, 5–6 days: 48.2, 7 days: 52.5. The quadratic trend was statistically significant in boys (p = 0.006) and girls (p<0.001).	NA	7
Petracci et al., 2013 [59]	Self-report The EQ-5D-Y (youth), 5-dimensional descriptive System: walking; looking after myself; doing usual activities; having pain or discomfort; and feeling worried, sad or unhappy. A Visual Analogue Scale (VAS), from 0 (worst imaginable health) to 100 (best imaginable health).	**Outcome:** Quantile of the VAS: 10th, 25th, 50th, 75th, and 90th quantiles **Method:** Quantile regression **Software used:** Stata 11.0	Children who exercised less than 2 hours/week had significantly lower scores in the10th quantile (6.9 points lower), the 25th quantile (3.8 points lower) and the 50th quantile (3.5 points lower) of the VAS relative to children who exercised more than 11 hours/week	NA	5
Spengler et al., 2013 [60]	Self-report The KINDL-R questionnaire	**Outcome:** Total score of the KINDL-R **Method:** Multiple linear regression **Software used:** SPSS 18.0	Adolescents who were less physically active experienced lower total HRQOL. The linear regression revealed a significant positive effect of PA in sports clubs on HRQOL (P = 0.039), and did not find a significant effect of PA during leisure time outside of sports clubs on HRQOL (β = 0.028; P = 0.522).	NA	5
Gopinath et al., 2012 [61]	Self-report. PedsQL 4.0. The HRQOL was measured at follow up	**Outcome:** Total and subscale scores **Method:** Linear regression, mixed model accounting for school clustering effect **Software used:** SAS 9.1	**(1) Cross-sectional associations:** Adolescents in higher level of PA (the highest versus the lowest tertile of total PA) had a significantly higher total PedsQL score (3.15-unit difference), higher scores in the physical summary (5.8-unit difference) and social (4.18-unit difference) domains. Adolescents in the highest tertile of outdoor PA had a higher total PedsQL score (2.19-unit difference), higher physical summary score (3.49-unit difference) and social domain (3.57 unit difference) than those in the lowest tertile. **(2) Temporal associations:** Adolescents with the highest level of PA relative to those in the lowest PA over the 5 years reported higher total scores (3.59 points difference), higher physical summary (7.21 points difference) and social scores (4.73 points difference).	**(1) Cross-sectional associations:** Adolescents in the highest versus lowest tertile of time spent in outdoor TV viewing showed lower total PedsQL score (3.51-unit difference). **(2) Temporal associations:** Youth in the highest tertile of total screen time during follow-up versus lowest tertile reported a lower total QOL score (75.87 vs. 82.21), lower scores in the physical summary (85.72 vs. 90.58), psychosocial summary (70.97 vs. 78.06), and emotional (66.98 vs. 75.31) and school (59.80 vs. 69.58) domains.	8
Wu et al., 2012 [62]	Self-report The EQ-5D-Y (youth)	**Outcome:** EQ-5D-Y dimension; VAS score **Method:** Multilevel logistic and multilevel linear regressions **Software used:** Stata 11.0	Children in physically inactive had significantly more HRQOL problems relative to their peers in physically active level on four of the five dimensions measured by the EQ-5D-Y except for the dimension of ‘walking’. Active children have significantly a higher VAS score than the inactive children (mean score difference = 5.1).	NA	6
Perry et al., 2012 [63]	Self-report The PedsQL 4.0	**Outcome:** Total score, physical health summary score **Method:** Linear regression **Software used:** SAS 9.2	In unadjusted models, active children had significantly better HRQOL (mean total score: 84.3 vs. 80.8; p < 0.05; physical function score: 91.5 vs. 85.6; p < 0.01) on the PedsQL relative to inactive children. Physical function subscale score remained significantly higher for active compared to inactive children (91.7 vs. 87.0; P <0.01) after adjusting for age, gender, race, family income, BMI and food security.	No significant difference in PedsQL scores was found between high and low screen time groups	5
Lacy et al., 2011 [64]	Self-report The PedsQL 4.0	**Outcome:** Total score. **Method:** Linear regression **Software used:** Stata 11.0	Participation in PA during the school day and after-school was associated with higher HRQOL (3.67-unit difference for boys, 4.25-unit difference for girls). The relationships remained after adjusting for weight status.	One hour increase in average screen time per day was significantly associated with decreased PedsQL total scores by 1.26 and 1.82 points among boys and girls, respectively. The PedsQL total score was 3.17 points, 4.01 points lower for boys and girls between adolescents with average screen time>2 h/day and ≤2 h/day.	7
Borras et al., 2011 [65]	Parent-report Child Health and Illness Profile-Child Edition /Parent Report Form (CHIP-CE/PRF). Four domains were used: Satisfaction with health, Physical comfort, Emotional comfort and Restricted activity.	**Outcome:** CHIP-CE/PRF domain scores **Method:** Pearson correlation **Software used:** SPSS 19.0	No significant association was found between PA and HRQOL	Screen time was associated with quality of life domains of physical comfort and restricted activity	3
Dalton et al., 2010 [66]	Self-report The PedsQL 4.0	**Outcome:** Total, and subscale scores **Method:** Linear regression **Software used:** Not reported	Higher number of physically active days per week was associated with higher HRQOL across multiple domains of PedsQL	Lower number of hours of screen time per day was associated with higher HRQOL across multiple domains of PedsQL	3
Hartmann et al., 2010 [67]	Parent-report German version of the Child Health Questionnaire (CHQ-PF50). 50 items, 13 domains, and were converted into a physical and a psychosocial summary scores.	**Outcome:** Physical and psychological health summary scores **Method:** Mixed linear regression model **Software used:** Not reported	PA intervention did not show improved physical QOL among the children. PA program had little positive influence (p<0.05) on psychosocial QOL in first graders. Psychosocial QOL score for intervention group of first grader: Pre-intervention: 53.2 (SD 5.9); Post-intervention: 53.6 (SD 6.0). Psychosocial QOL score for control group of first grader: Pre-intervention: 53.3 (SD 6.0); Post-intervention: 51.7 (SD 7.3).	NA	7
Kriemler et al., 2010 [68]	Parent-report CHQ-PF50	**Outcome:** Physical and psychological health summary scores **Method:** Mixed linear regression model **Software used:** Not reported	School-based PA program did not show improved QOL among the children	NA	7
Boyle et al., 2010 [69]	Self-report PedsQL 4.0, EQ-5D-Y, a Visual Analogue (VAS) on 0 (worst possible health) to 100 (best possible health) scale	**Outcome:** PedsQL total, physical and psychosocial health summary scores, subscale scores, EQ-5D VAS, EQ-5D utility. **Method:** Linear regression **Software used:** SPSS 14.0	No statistically significant relationship between PA and QOL measured by the PedsQL and the EQ-5D utility score. Children achieving the 60 min of PA per day recommendations reported better EQ-5D-Y VAS scores than those who did not achieve the recommendations.	NA	6
Gordia et al., 2010 [70]	Self-report The QOL was measured using the WHOQOL-Bref, which includes 26 questions. The WHOQOL-Bref score ranges from 1 to 100, with higher scores indicating better QOL. The study used data of the psychological domain.	**Outcome:** Psychological domain **Method:** Logistic regression **Software used:** Not reported	Less active students were more likely (OR = 1.90, 95% CI: 1.16–3.10) to have a negative perception on the psychological domain of quality of life	NA	5
Herman et al., 2010 [71]	Self-report The Medical Outcomes Study Short Form 36 (SF-36). 36 questions, including 8 domains: physical function (PF), role physical (RE), bodily pain (BP), general health (GH), vitality (VT), social functioning (SF), role emotional (RE), and mental health (MH). Domains are summed to physical and mental component summary scores (PCS and MCS). Domain scores range from 0 to 100, with higher scores indicating better HRQL.	**Outcome:** Domains, PCS and MCS scores **Method:** Logistic regression **Software used:** Not reported	No significant association between PA and QOL at any domain or the summary score of SF-36 was observed in the logistic regression	NA	5
Mathers et al., 2009 [72]	Self-report The PedsQL 4.0, and the KIDSCREEN-10. Higher scores indicate higher HRQoL.	**Outcome:** The continuous outcomes of PedsQL 4.0 total score and KIDSCREEN-10 **Method:** Linear regression **Software used:** Stata 10.0	NA	Adolescents who spent more time using electronic media (≥255 minutes) had lower PedsQL total score; lower KIDSCREEN score than those who spent the least amount of time (<121minutes). High levels of video game use were associated with poorer HRQOL.	5
Sánchez-López et al., 2009 [73]	Self-report Child Health and Illness Profile-Child Edition (CHIP-CE), 45 items, five dimensions: satisfaction, comfort, resilience, risk avoidance and achievement. Higher scores represent better health.	**Outcome:** CHIP-CE dimensions’ scores **Method:** Analysis of co-variance (ANCOVA) **Software used:** SPSS 15.0	Active children had higher mean dimension scores than sedentary children, except for risk avoidance. Among active children, girls had significantly higher scores on the resilience, risk avoidance and achievement dimensions than boys. Sedentary girls showed higher scores on the risk avoidance and achievement dimensions, and sedentary boys had higher scores on the comfort dimension.	NA	6
Wang et al., 2008 [74]	Self-report Japanese version of the COOP charts. The chart ‘‘Quality of life” question: ‘‘How have things been going for you during the past 4 weeks?” Five response categories: very well, pretty good, good and bad parts about equal, pretty bad, very bad. QOL was classified as ‘good QOL’ by combining the ‘very good’ and ‘good’ responses into one group, and ‘poor QOL’ by combining the ‘about equal, pretty bad, and very bad’.	**Outcome:** QOL dichotomous outcome **Method:** Multiple logistic regression **Software used:** SPSS 11.5	Less active children relative to active children had lower QOL in early adolescents (OR = 1.51, P = 0.016)	NA	7
Chen X et al., 2005a [75]	Self-report Japanese version of the COOP Charts. Subjects rated as ‘very well’ or ‘pretty good’ for the QOL question were considered to have ‘good QOL’ and the remainder (about equal, pretty bad, and very bad) was classified as having ‘poor QOL’.	**Outcome:** QOL dichotomous outcome **Method:** Multiple logistic regression **Software used:** SPSS 10.0	Compared with children participating in PA ‘very often’ at baseline, those participating in PA ‘seldom’ or ‘almost never’ were more likely to have poor QOL (OR = 1.61, 95% CI: 1.42–1.84; OR = 2.06, 95% CI:1.39–3.05) at the follow-up. A dose-response effect was showed. Relative to those who maintained higher PA as ‘often’, children who changed from ‘often’ to ‘seldom’ and who remained ‘seldom’ were more likely to have poor QOL at follow-up (OR = 2.10, 95% CI: 1.84–2.39; OR = 2.21, 95% CI: 1.88–2.59).	Compared to children with <2 hours of television viewing, those watching TV≥3 hours had a high OR of 1.24, 95% CI: 1.10–1.39 for poor QOL at follow-up. A dose-response effect was observed.	7
Chen X et al., 2005b [76]	Self-report Japanese version of the COOP Charts. Nine domains: physical fitness, feelings, daily activities, social activities, pain, overall health, change in health, social support, and quality of life.	**Outcome:** QOL dichotomous outcome **Method:** Multiple logistic regression **Software used:** SPSS 10.0	Children who were in low frequency of PA had poor QOL compared with their active peers. Dose-response relation between PA and poor quality of life (reference for PA: very often, multivariate model) was observed: Quite often: OR = 1.63, 95% CI: 1.43–1.82; Seldom: OR = 2.36, 95% CI: 2.09–2.94; Almost never: OR = 4.39, 95% CI: 3.19–6.03.	Children with longer TV viewing were more likely to have poor QOL. Dose-response relation between QOL and time spent in watching TV (reference: <2 hours, univariate analysis): 2–3 h: OR = 1.17, 95% CI: 1.05–1.3; 3–4 h: OR = 1.25, 95% CI: 1.07–1.44; >4 h: OR = 1.34, 95% CI: 1.14–1.59.	6

**Abbreviations:** HRQOL: health-related quality of life; PedsQL 4.0: the Pediatric Quality of Life Inventory; EQ-5D: European Quality of Life 5 Dimension measure; EQ-5D-Y: European Quality of Life 5 Dimension measure for youth; CHIP-CE: Child Health and Illness Profile-Child Edition; CHQ-PF50: Child Health Questionnaire Parent-completed Form; SG: standard gambling; BWS: best worst scaling; SBM: screen-based media; OR: odds ratio; CI: confidence interval; NA: not applicable.

### Risk of bias assessment

The average score of risk of bias among all included studies was 5.71 (SD = 1.47). The risk of bias score ranged between 3 and 8 for the individual studies. A total of 20 studies were scored as moderate risk of bias (n = 14) and high risk of bias (n = 6). Eleven studies were rated as low risk of bias (Table 2). The common reasons for the low quality included small sample size, lack of control for important confounders, inadequate statistical methods, and non-response bias. The overal evidence of the study quality was low since most of the studies were based on cross-sectional design with only a few longitudinal studies and RCTs.

### Associations between physical activity and HRQOL among children and adolescents

#### Findings of the cross-sectional studies

Table 2 presents the fingdings for the association between PA, sedentary behavior and HRQOL. Of the 21 cross-sectional studies that examined the relationship between PA and HRQOL among children and adolescents, 19 studies found that children and adolescents in a higher level of PA compared with those in the lower level of PA had significantly higher HRQOL. Finne et al.(2013) found in a representative sample of German children and adolescents aged 11–17 years that a higher frequency of PA was related to higher HRQOL with small to moderate effect sizes for daily PA relative to no regular PA in both boys and girls. Gopinath et al.(2012) reported that adolescents (n = 1,094) in the highest tertile of total PA had significantly 3.15 points higher in total PedsQL scores compared with those in the lowest tertile of total PA [61]. Similarly, adolescents in the highest tertile relative to the lowest tertile of total PA had higher scores in the physical summary (5.8 points difference) and social (4.18 points difference) domains [61]. Lacy et al.(2011) found among a large sample of Australian adolescents (n = 3,040) that higher levels of PA were associated with higher HRQOL for both boys and girls, and the relationship remained after adjusting for weight status [64].

A dose-response relation between PA and HRQOL was reported by three studies with large samples [57,58,76]. A significant linear trend between PA and HRQOL was observed in a dose-response manner for most subscales of the PedsQL 4.0 in the study by Finne et al. [57]. Galán et al.(2013) reported a dose-response association between moderate to vigorous physical activity (MVPA) and HRQOL in a national sample of Spanish children and adolescents (n = 16,560, aged 11–18 years), where an increase in PA levels was related to additional improvements in HRQOL [58]. One Japanese study (Chen et al.,2005b) showed a dose-response relation between PA and HRQOL, where children who engaged in lower frequency of PA had a higher odds of experiencing poor HRQOL [76].

Three studies used the EQ-5D-Y, a recent developed HRQOL measure for children and youth between 8–18 years that has been previously validated [77,78]. Petracci et al.(2013) used quantile regression in their study and found that children who exercised less than 2 hours a week had significantly lower scores of the EQ-5D-Y VAS relative to children who exercised more than 11 hours a week [59]. Wu et al. (2012) observed in a population-based sample of Canadian grade five students (n = 3,421) that physically active children reported significantly less HRQOL problems (based on the adjusted odds ratio) relative to their peers who were physically inactive on four of the five EQ-5D-Y dimensions: ‘looking after myself’, ‘doing usual activities’, ‘having pain or discomfort’, and ‘feeling worried, sad or unhappy’. Children who were physically active had a significanlty higher VAS score (difference = 4.49 points) compared with those peers who were physically inactive [62]. The observed relations between PA and HRQOL were independent of the potential confounding effect of the socio-demographic factors (gender, parental education level and family income), diet quality and weight status. Boyle et al. (2010) observed that children who achieved the recommendation of 60 minute PA per day had significantly better EQ-5D-Y VAS scores than those who did not achieve the recommendation [69].

One study did not observe a significant relation between PA and HRQOL, and the study was rated as having a high risk of bias due to small sample and reliance only on the analysis of Pearson correlation [65]. Another study observed a significant correlation between PA and HRQOL based on Pearson correlation, but not in multiple regression analysis [56].

#### Findings of the longitudinal studies and the randomised controlled trials

Of the seven studies that examined longitudinal associations between PA and HRQOL, six studies [48,53,54,61,74,75] observed a significant effect among children and adolescents. Omorou et al.(2016) found that the cumulative level of high PA was associated with high HRQOL at 2-year follow up among adolescents (n = 1,445) in all four dimensions of the Duke Health Profile: physical, mental, social and general health [48]. Vella,et al.(2014) reported that children who maintained participation in sports throughout the 2-year follow up had better HRQOL at follow-up than children who did not participate in sports, dropped out of sports, and commenced participation after the baseline [54].The magnitude of differences in total PedsQL score between sport participants and nonparticipants was approximately 5 units, greater than the minimum clinically meaningful difference of 4.5 units on PedsQL [54]. Gopinath et al.(2012) examined temporal relations between PA and HRQOL in adolescents [61]. The result showed that adolescents who remained in the highest level of PA over the 5 years of follow up compared with those in the lowest level of total PA had higher scores in the PedsQL total score (P = 0.04), physical summary (P = 0.0001), and social (P = 0.02) domains. Two Japanese cohort studies evaluated longitudinal relations between PA and HRQOL among children [74,75]. Wang et al. (2008) found that children who were less active in early childhood (aged 3 years) had a greater odds (OR = 1.51, p = 0.016) of having lower QOL in their early adolescents (first year of junior high school study) compared with the peers who were active in early childhood [74]. Chen et al. (2005a) reported in a cohort of 7,794 children aged 9–10 years that children with higher frequency of PA (‘very often’) relative to lower frequency of PA (‘seldom’ or ‘almost never’) at baseline survey (1999) experienced better QOL after 3 years of follow up (2002) [75]. In comparison with children who maintained higher PA as ‘often’, children who changed from ‘often’ to ‘seldom’ and who remained ‘seldom’ were more likely to have poor QOL (OR = 2.10, 95% CI:1.84–2.39; OR = 2.21, 95% CI:1.88–2.59) [75]. The longitudinal study in Canada did not find a sigificant effect of physical activity in youth on the HRQOL 22 years later in adulthood using a subgroup of participants aged 7–18 years at baseline from a Canadian population-based survey [71].

Of the three cluster-randomized controlled trials, one study assessed the effect of a school-community program on HRQOL in adolescent girls in Australia [51], and two cluster-RCTs evaluated the effect of a school-based PA program on HRQOL in school children in Switzerland [67,68]. Casey et al. (2014) found that the intervention program positively influenced quality of life of adolescent girls [51]. The other two RCTs did not show improved physical QOL among the children, and only a little positive influence (p<0.05) of the program was observed on psychosocial QOL in first grade students [67,68].

### Associations between sedentary behavior and HRQOL among children and adolescents

#### Findings of the cross-sectional studies

Of the included studies, 17 studies assessed the association between sedentary behavior and HRQOL among children and adolescents, including 13 cross-sectional studies and 4 longitudinal studies (Table 2). Most of the cross-sectional studies reported a significant association between sedentary behavior and poor HRQOL. The findings for the association are consistent across different types of sedentary behaviors, such as television viewing, using computers, playing video games, reading and doing homework and screen time measured by an accelerometer. Two cross-sectional studies did not observe a significant association between screen time and HRQOL, and these studies had relatively small samples (n = 156 and n = 371) that may compromise adequate statistical power [50,63]. Finne et al.(2013) reported a dose-response relation between time spent on screen-based media use and HRQOL [57].

Similarly to the effect of PA on the domains of HRQOL, sedentary behavior was linked to the multiple domains of HRQOL in childhood and adolescence. Children and adolescents who spent more time in sedentary activities reported lower HRQOL in physical, mental and psychosocial health, school functioning, and general health domains [47,57,65,66]. Chen G,et al. (2014) and Xu,et al. (2014) found that longer time spent on TV viewing, playing computers or video games, or doing homework was associated with a lower utility score measured by the Child Health Utility 9D [52,55].

#### Findings of the longitudinal studies

All four studies with the longitudinal design found consistently that more time spent on sedentary activities (television viewing, use of computers or video games, telephones) correlated with reduced HRQOL [48,53,61,75]. Omorou et al.and Gopinath et al. observed that greater screen time during follow-up was related to lower scores in several domains of QOL including physical, psychosocial, mental, emotional and school domains [48,61]. In addition, Chen X, et al.(2005a) reported that there is a dose-response relation between screen time and HRQOL, where children who engaged in longer screen time were more likely to have poor HRQOL at follow up [75].

### Findings from the meta-analysis

**Fig 2:**
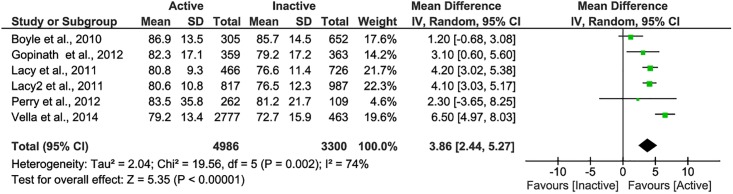
Forest plot of the mean difference in PedsQL total scores between physically active and inactive children and adolescents. Study by Lacy et al. 2011 has two groups: adolescents <15 years (Lacy et al. 2011); adolescents ≥15 years (Lacy2 et al. 2011).

Meta-analysis was performed for five observational studies using the PedsQL measure [54,61,63,64,69]. There was a significant difference in total PedsQL scores (mean difference = 3.86, 95% CI: 2.44–5.27, P<0.01) between inactive and active children and adolescents (Fig 2). A higher level of PA was associated with an increased PedsQL total score.

**Fig 3:**
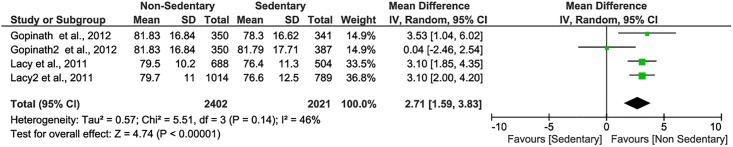
Forest plot of the mean difference in PedsQL total scores between screen time of sedentary behavior. Study by Gopinath et al. has two groups by screen time: the first group (Gopinath, et al. 2012) indicates 2nd tertile of screen time 2.57–3.86 hours relative to the non-sedentary group (≤2.5 hours/day); the second group (Gopinath2, et al. 2012) indicates 3rd tertile of screen time ≥3.93 hours relative to the non-sedentary group. Study by Lacy et al. has two groups by age of the adolescents: <15 years in the first group (Lacy et al., 2011); and ≥15 years in the second group (Lacy2 et al., 2011); the non-sedentary (reference) group is defined as total daily screen time≤2.0 hours/day.

Pooled analysis for the four subgroups of the two studies indicated a significant overall difference in total PedsQL scores (mean difference = 2.71, 95% CI: 1.59–3.83, P<0.01) between sedentary (>2 hours/day) and non-sedentary (≤2 hours/day) children and adolescents [61,64], suggesting that more time of being sedentary is related to worse HRQOL (Fig 3).

## Discussion

This review synthesized the relationship between physical activity, sedentary behavior and health-related quality of life among the general population of healthy children and adolescents. We found the evidence that elevated levels of physical activity are associated with higher HRQOL and sedentary behavior is inversely related to HRQOL among children and adolescents. Physical activity and sedentary behavior have significant effects on multiple physical, mental and psychosocial domains of HRQOL.

The results from cross-sectional studies revealed that children and adolescents who participated in higher levels of physical activities had better HRQOL. The association between PA and HRQOL is consistent irrespective of weight status, age, sex, and socio-economic characteristics. The findings are also in agreement with the previous observation in general adult populations showing that PA has a positive influence on HRQOL [19].

We found some evidence in this review for a dose-response effect between PA and HRQOL. For example, Finne et al. reported a linear trend between frequency of PA and PedsQL total and subscale scores, indicating a dose-response relation in both genders [57]. Galán revealed an increasing dose-response relationship between moderate to vigorous physical activity and HRQOL in both genders of adolescents, with an observation of both linear and quadratic trends [58]. The dose-response association between frequency of PA and HRQOL was also showed in a Japanese cross-sectional study in children [76]. Yet more studies are needed to confirm the association and whether the relationship of HRQOL with PA is linear or nonlinear since the finding was primarily based on cross-sectional studies and previous studies have also reported nonlinear associations between PA and HRQOL among adults [19,79].

The relationship between physical activity and HRQOL was observed for both total QOL score and subscale scores including physical, psychological and social subscales [48,49,51,53,54,57,61–64,66,67]. These results lend a support to a number of previous studies demonstrating that children and adolescents who maintain an active lifestyle exhibit better physical and better psychosocial health [2,3,5,42].

Longitudinal observational studies support a positive asscoiation between PA and HRQOL. Six studies found a significant longitudinal or temporal association between PA and HRQOL, and most of these studies analysed large samples of children and adolesents [48,53,54,61,74,75]. A dose-response effect of PA on HRQOL was observed in a cohort of children with a three years follow up. However, the HRQOL data were not available at baseline in some cohort studies, and thus limiting the ability to examine changes in QOL outcomes in relation to changes in PA and sedentary behavior [61,74,75]. Further, it did not permit to account for the impact of baseline HRQOL on the HRQOL at follow up. Despite the longitudinal and some intervention studies demonstrated that PA predicts future HRQOL, we can not conclude a causal effect of PA on QOL given that the number of longitudinal and RCT studies is small and one study also found that HRQOL predicts PA later [48].

The studies that assessed the effect of sedentary behavior on HRQOL support the finding that longer sedentary time is connected with decreased HRQOL in children and adolescents. The association between sedentary behavior and HRQOL is independent of the PA, gender, age and body weight among children and adolescents. Watching TV, use of computers and playing video games for more than two hours a day are significantly associated with lower HRQOL [52,57,61,64,72,76]. The findings from the longitudinal studies showed that children and adolescents who spent greater time on sedentary activities during follow-up experienced worse HRQOL at follow up [48,53,61,75], suggesting a negative effect of sedentary behavior for future HRQOL. A dose-response relation between screen time and adverse QOL was reported in three observational studies [57,75,76]. Children and youth who spent excessive amount of time on sedentary behaviors reported lower HRQOL in physical, mental and psychosocial health domains [47,48,57,61,65,66]. For example, Finne et al., 2013 reported that there was a dose-response relation for most subscales of the KINDL-R except for the family domain among girls, and a dose-response relation for physical well-being and school domains among boys [57]. The evidence of the adverse impact of sedentary behavior on mental/psychosocial health is consistent with a number of previous studies that focused on the outcomes of mental health in school-aged children and adolescents [25].

Due to the methodological heterogeneities in the measurement of PA, sedentary behavior and HRQOl, we highlighted the results in this review from qualitative synthesis. We performed meta-analyses for five studies that used the same QOL measure of PedsQL, and similar assessment methods for PA and sedentary behavior. The findings from the pooled analysis support a beneficial effect of PA and a detrimental effect of sedentary behaviors for HRQOL among children and youth. Future research would warrant to examine the magnitude of the difference in QOL from meta-analysis by inclusion of more studies that utilize similar measures of PA, sedentary behavior and HRQOL. Specifically, consistent measures for physical activity and sedentary behavior across studies are required to assess intensity and frequency of the health-related behaviors to facilitate the inter-group comparisons.

The strength of the associations between HRQOL and PA or sedentary behavior in this study was largely based on judgement from statistical significance of the difference in QOL outcomes. When comparing the difference in HRQOL values across groups by PA and sedentary behavior, it is also important to examine the extent of the score difference (e.g.,effect size) in order to determine the minimally important difference (MID) of QOL scores [80] as the MID signifies a meaningful difference that has clinical and practical importance for public health and health interventions. Based on Cohen’s criteria, effect size for HRQOL is defined as small (0.2), moderate (0.5), and large (0.8) in the magnitude for differences between groups [81]. In our review, the MID or effect size for group comparisons in QOL was discussed or reported in eleven studies [48,51,52,54,55,57,58,62,67–69]. The MID criterion varies with HRQOL instruments, and is more frequently used in clinical settings to compare HRQOL among patients with chronic disease conditions than in healthy populations [80]. For example, the study by Omorou et al. indicated that the difference in HRQOL scores between active and inactive living adolescents during two years of follow-up was close to or greater than 5 points (considered as clinically meaningful difference) for all the four dimensions of the Duke Health Profile: physical, mental, social and general health [48]. Vella,et al.(2014) reported that children who maintained participation in sports throughout a 2-year follow up period had an approximately 5 units higher total PedsQL score at follow-up than children who did not participate in sports [54]. A 4.5-unit difference in HRQOL between group comparisons was considered as the MID value for the PedsQL [82].

There are several strengths of this review. The present study is the first to review the associations between physical activity, sedentary behavior and HRQOL among studies using population-based samples of children and adolescents. We conducted a comprehensive literature search and did rigorous selection and assessments for the eligible studies using predefined inclusion and exclusion criteria. We included both PA and sedentary behavior as the predictor variables for HRQOL which allowed us to examine the independent effect of the two health-related behaviors on HRQOL. The majority of the included studies analysed data from large samples of children and adolescents. This offered good opportunities for the analysis to adjust for the effect of important confounders such as body weight, age, gender, parental education and household income. Thus the systematic review contributes to the existing literature for the associations between PA, sedentary behavior and HRQOL which are independent of body weight and socio-demographic characteristics of children and youth. Consistent associations between PA, sedentary behavior and HRQOL were reported across studies in different countries, suggesting that these results are robust. The outcome was measured by a variety of generic multi-dimensional HRQOL measures that have been validated for pediatric and youth populations, allowing for investigation of the effect of PA, sedentary behaviors on a single dimension of QOL as well as the overall QOL. Most primary studies applied multivariable regression analyses and some used multilevel regressions to account for hierarchical feature of school data, and thus providing appropriate and rigorous statistical methods for parameter estimates.

Limitations of this review also deserve to be clarified. Most of the included studies are cross-sectional, and very few longitudinal and intervention studies are identified. Informative conclusion can not be made about causal relationship between PA, sedentary behavior and HRQOL. PA and sedentary behavior assessments in the included studies were largely based on child and youth self-report or parent-report, and thus may have affected the measurement error. The use of objective measures of PA (e.g., pedometer) and sedentary behavior (e.g., accelerometer, inclinometer or screen use monitor) is needed to make more accurate assessments of these behaviors [83]. In addition, the citation and abstract screening for selection of the eligible studies was done by a single reviewer (XYW) rather than use of two reviewers. However, we made alternative efforts to ensure the study selection is intensive, rigorous, complete and accurate to meet the study goal. These work included the use of predefined inclusion and exclusion criteria, the evaluation of the full-text articles of potetial eligible studies by the two reviewers (XYW, LHH), and comprehensive screening for PubMed-related articles and reference lists of the relevant studies to identify other eligible studies.

Children and adolescents spend more time engaging in sedentary activities than a decade ago due to the increasing use of screen-based electronic devices (e.g., smart phones, laptops) and widespread accessibility to the internet [84]. Many countries have so far developed physical activity and/or sedentary behavior guidelines for school-age children and adolescents in order to improve their health [85–87]. WHO suggests that children and youth aged 5–17 years old should accumulate at least 60 minutes of moderate to vigorous-intensity of physical activity everyday [88]. Yet majority of the young people in many countries do not meet the recommendations of PA levels [89–93]. HRQOL comprises multifaceted aspects of health. The importance of PA and sedentary behavior for QOL is that promoting PA and decreasing sedentary behaviors among young people may benefit not only to a specific health condition (e.g., obesity) but also to their mental health and overal health status. Therefore, it is worth to further investigate to determine the appropriate amount of PA, time spent in sedentary behaviors for HRQOL improvement. For exampple, whether a threshold exists for having no additional benefits on total or a dimension of HRQOL when PA or sedentary time exceeds a certain amount. As PA and sedentary behavior can be measured and expressed in different types (e.g., objective or subjective), different aspects (e.g., leisure time PA, TV or computer use) and different units (e.g., frequency, intensity and duration), future research is needed to investigate which types or aspects of physical activity and sedentary behavior would be the most significant contributors for QOL. In the meantime, to facilitate comparisons using quantitative synthesis of different studies, standardized measures as well as the objective measures of these health-related behavioral indicators for QOL are needed. Future studies are also needed to examine prospectively how changes in these behaviors affect changes in HRQOL among children and adolescents. This will provide useful information for evalations of the effectiveness health promotion programs targeting to modify unfavorable inactive and sedentary behaviors.

## Conclusions

The present review has found the evidence that a higher level of physical activity and less time spent on sedentary behavior are associated with increased health-related quality of life among the general population of children and adolescents. Future research is needed to identify potential causal mechanisms for these relationships. More longitudinal and cluster-randomized controlled trials are required to assess the dose-response effect of physical activity and sedentary behavior on health-related quality of life among children and adolescents. This will help justify school health intervention efforts promoting active lifestyle, reducing sedentary behaviors to enhance quality of life of the young population. The findings in this review may be used as evidence to inform primary prevention and public health policy for promoting the health of children and youth.

## Supporting information

S1 Table Literature search strategies for the databases of MEDLINE, EMBASE and PSYCINFO (Table A-Table C). (DOC)

S2 Table PRISMA checklist. (DOC)

## References

[1] Janssen I, Leblanc AG. Systematic review of the health benefits of physical activity and fitness in school-aged children and youth. Int J Behav Nutr Phys Act. 2010;7:40 doi: 10.1186/1479-5868-7-40 20459784 10.1186/1479-5868-7-40 PMC2885312

[2] Iannotti RJ, Janssen I, Haug E, Kololo H, Annaheim B, Borraccino A, et al Interrelationships of adolescent physical activity, screen-based sedentary behaviour, and social and psychological health. Int J Public Health. 2009;54 Suppl 2:191–198. 19639256 10.1007/s00038-009-5410-z PMC2732761

[3] Ussher MH, Owen CG, Cook DG, Whincup PH. The relationship between physical activity, sedentary behaviour and psychological well being among adolescents. Soc Psychiatry Psychiatr Epidemiol. 2007;42(10):851–856. doi: 10.1007/s00127-007-0232-x 17639309 10.1007/s00127-007-0232-x

[4] Hallal PC, Victora CG, Azevedo MR, Wells JC. Adolescent physical activity and health: a systematic review. Sports Med. 2006;36(12):1019–1030. 17123326 10.2165/00007256-200636120-00003

[5] Iannotti RJ, Kogan MD, Janssen I, Boyce WF. Patterns of adolescent physical activity, screen-based media use, and positive and negative health indicators in the U.S. and Canada. J Adolesc Health. 2009;44(5):493–499. doi: 10.1016/j.jadohealth.2008.10.142 19380098 10.1016/j.jadohealth.2008.10.142 PMC2705990

[6] Prentice-Dunn H, Prentice-Dunn S. Physical activity, sedentary behavior, and childhood obesity: A review of cross-sectional studies. Psychol Health Med. 2012;17(3):255–273. doi: 10.1080/13548506.2011.608806 21995842 10.1080/13548506.2011.608806

[7] Leung MM, Agaronov A, Grytsenko K, Yeh MC. Intervening to reduce sedentary behaviors and childhood obesity among school-age youth: A systematic review of randomized trials. J Obes. 2012;2012:685430 doi: 10.1155/2012/685430 22132321 10.1155/2012/685430 PMC3202121

[8] Veugelers PJ, Fitzgerald AL. Effectiveness of school programs in preventing childhood obesity: a multilevel comparison. Am J Public Health. 2005;95(3):432–435. doi: 10.2105/AJPH.2004.045898 15727972 10.2105/AJPH.2004.045898 PMC1449197

[9] Brown T, Summerbell C. Systematic review of school-based interventions that focus on changing dietary intake and physical activity levels to prevent childhood obesity: an update to the obesity guidance produced by the National Institute for Health and Clinical Excellence. Obes Rev. 2009;10(1):110–141. doi: 10.1111/j.1467-789X.2008.00515.x 18673306 10.1111/j.1467-789X.2008.00515.x

[10] Page AS, Cooper AR, Griew P, Jago R. Children's screen viewing is related to psychological difficulties irrespective of physical activity. Pediatrics. 2010;126(5):e1011–1017. doi: 10.1542/peds.2010-1154 20937661 10.1542/peds.2010-1154

[11] Costigan SA, Barnett L, Plotnikoff RC, Lubans DR. The health indicators associated with screen-based sedentary behavior among adolescent girls: a systematic review. J Adolesc Health. 2013;52(4):382–392. doi: 10.1016/j.jadohealth.2012.07.018 23299000 10.1016/j.jadohealth.2012.07.018

[12] Tremblay MS, LeBlanc AG, Kho ME, Saunders TJ, Larouche R, Colley RC, et al Systematic review of sedentary behaviour and health indicators in school-aged children and youth. Int J Behav Nutr Phys Act. 2011;8:98 doi: 10.1186/1479-5868-8-98 21936895 10.1186/1479-5868-8-98 PMC3186735

[13] Solans M, Pane S, Estrada MD, Serra-Sutton V, Berra S, Herdman M, et al Health-related quality of life measurement in children and adolescents: a systematic review of generic and disease-specific instruments. Value Health. 2008;11(4):742–764. doi: 10.1111/j.1524-4733.2007.00293.x 18179668 10.1111/j.1524-4733.2007.00293.x

[14] World Health Organization. Constitution of the World Health Organization. Geneva: World Health Organization, 1948.

[15] Yackobovitch-Gavan M, Nagelberg N, Phillip M, Ashkenazi-Hoffnung L, Hershkovitz E, Shalitin S. The influence of diet and/or exercise and parental compliance on health-related quality of life in obese children. Nutr Res. 2009;29(6):397–404. doi: 10.1016/j.nutres.2009.05.007 19628106 10.1016/j.nutres.2009.05.007

[16] Shoup JA, Gattshall M, Dandamudi P, Estabrooks P. Physical activity, quality of life, and weight status in overweight children. Qual Life Res. 2008;17(3):407–412. doi: 10.1007/s11136-008-9312-y 18293100 10.1007/s11136-008-9312-y

[17] Crosbie A. The effect of physical training in children with asthma on pulmonary function, aerobic capacity and health-related quality of life: a systematic review of randomized control trials. Pediatr Exerc Sci. 2012;24(3):472–489. 22971562 10.1123/pes.24.3.472

[18] Paxton RJ, Jones LW, Rosoff PM, Bonner M, Ater JL, Demark-Wahnefried W. Associations between leisure-time physical activity and health-related quality of life among adolescent and adult survivors of childhood cancers. Psychooncology. 2010;19(9):997–1003. doi: 10.1002/pon.1654 19918964 10.1002/pon.1654 PMC2888632

[19] Bize R, Johnson JA, Plotnikoff RC. Physical activity level and health-related quality of life in the general adult population: a systematic review. Prev Med. 2007;45(6):401–415. doi: 10.1016/j.ypmed.2007.07.017 17707498 10.1016/j.ypmed.2007.07.017

[20] Davies CA, Vandelanotte C, Duncan MJ, van Uffelen JG. Associations of physical activity and screen-time on health related quality of life in adults. Prev Med. 2012;55(1):46–49. doi: 10.1016/j.ypmed.2012.05.003 22588226 10.1016/j.ypmed.2012.05.003

[21] Caspersen CJ, Powell KE, Christenson GM. Physical activity, exercise, and physical fitness: definitions and distinctions for health-related research. Public Health Rep. 1985;100(2):126–131. 3920711 PMC1424733

[22] Tremblay MS, Aubert S, Barnes JD, Saunders TJ, Carson V, Latimer-Cheung AE, et al; SBRN Terminology Consensus Project Participants. Sedentary Behavior Research Network (SBRN)—Terminology Consensus Project process and outcome. Int J Behav Nutr Phys Act. 2017;14(1):75 doi: 10.1186/s12966-017-0525-8 28599680 10.1186/s12966-017-0525-8 PMC5466781

[23] Network SBR. Letter to the Editor: Standardized use of the terms "sedentary" and "sedentary behaviours". Appl Physiol Nutr Metab. 2012;37(3):540–542. doi: 10.1139/h2012-024 22540258 10.1139/h2012-024

[24] Wells GA, Shea B, O'Connell D, Peterson J, Welch V, Losos M, et al. The Newcastle-Ottawa Scale (NOS) for assessing the quality of nonrandomized studies in meta-analyses. http://www.ohri.ca/programs/clinical_epidemiology/oxford.asp. Accessed on May 21, 2017.

[25] Suchert V, Hanewinkel R, Isensee B. Sedentary behavior and indicators of mental health in school-aged children and adolescents: A systematic review. Prev Med. 2015;76:48–57. doi: 10.1016/j.ypmed.2015.03.026 25895839 10.1016/j.ypmed.2015.03.026

[26] Higgins JPT, Thompson SG: Quantifying heterogeneity in a meta-analysis. Stat Med. 2002;21:1539–1558. doi: 10.1002/sim.1186 12111919 10.1002/sim.1186

[27] Moher D, Liberati A, Tetzlaff J, Altman DG. Preferred reporting items for systematic reviews and meta-analyses: the PRISMA Statement. J Clin Epidemiol. 2009;62(10):1006–1012. doi: 10.1016/j.jclinepi.2009.06.005 19631508 10.1016/j.jclinepi.2009.06.005

[28] Dumuid D, Olds T, Lewis LK, Martin-Fernández JA, Katzmarzyk PT, Barreira T, et al International Study of Childhood Obesity, Lifestyle and the Environment (ISCOLE) research group. Health-related quality of life and lifestyle behavior clusters in school-aged children from 12 countries. J Pediatr. 2017;183:178–183.e2 doi: 10.1016/j.jpeds.2016.12.048 28081885 10.1016/j.jpeds.2016.12.048

[29] Liu J, Sekine M, Tatsuse T, Fujimura Y, Hamanishi S, Lu F, Zheng X. Outdoor physical activity and its relation with self-reported health in Japanese children: results from the Toyama birth cohort study. Child Care Health Dev. 2015;41(6):920–927. doi: 10.1111/cch.12262 26073535 10.1111/cch.12262

[30] Padilla-Moledo C, Castro-Piñero J, Ortega FB, Pulido-Martos M, Sjöström M, Ruiz JR. Television viewing, psychological positive health, health complaints and health risk behaviors in Spanish children and adolescents. J Sports Med Phys Fitness. 2015;55(6):675–683. 25895471

[31] Smart JE, Cumming SP, Sherar LB, Standage M, Neville H, Malina RM. Maturity associated variance in physical activity and health-related quality of life in adolescent females: a mediated effects model. J Phys Act Health. 2012;9(1):86–95. 22232510 10.1123/jpah.9.1.86

[32] Kalak N, Gerber M, Kirov R, Mikoteit T, Yordanova J, Pühse U, et al Daily morning running for 3 weeks improved sleep and psychological functioning in healthy adolescents compared with controls. J Adolesc Health. 2012;51(6):615–622. doi: 10.1016/j.jadohealth.2012.02.020 23174473 10.1016/j.jadohealth.2012.02.020

[33] Gunnell KE, Brunet J, Sabiston C, Bélanger M. Linking psychological need satisfaction and physical activity to dimensions of health-related quality of life during adolescence: A test of direct, reciprocal, and mediating effects. J Sport Exerc Psychol. 2016;38(4):367–380. doi: 10.1123/jsep.2015-0325 27736288 10.1123/jsep.2015-0325

[34] Pindus DM, Cumming SP, Sherar LB, Gammon C, Coelho e Silva M, Malina RM. Maturity-associated variation in physical activity and health-related quality of life in British adolescent girls: moderating effects of peer acceptance. Int J Behav Med. 2014;21(5):757–766. 25356455 10.1007/s12529-013-9344-8

[35] Quaresma AM, Palmeira AL, Martins SS, Minderico CS, Sardinha LB. Effect of a school-based intervention on physical activity and quality of life through serial mediation of social support and exercise motivation: the PESSOA program. Health Educ Res. 2014;29(6):906–917. doi: 10.1093/her/cyu056 25274722 10.1093/her/cyu056

[36] Conry MC, Morgan K, Curry P, McGee H, Harrington J, Ward M, et al The clustering of health behaviours in Ireland and their relationship with mental health, self-rated health and quality of life. BMC Public Health. 2011;11:692 doi: 10.1186/1471-2458-11-692 21896196 10.1186/1471-2458-11-692 PMC3187756

[37] Guallar-Castillón P, Bayán-Bravo A, León-Muñoz LM, Balboa-Castillo T, López-García E, Gutierrez-Fisac JL, et al The association of major patterns of physical activity, sedentary behavior and sleep with health-related quality of life: a cohort study. Prev Med. 2014;67:248–254. doi: 10.1016/j.ypmed.2014.08.015 25138382 10.1016/j.ypmed.2014.08.015

[38] Saunders TJ, Gray CE, Poitras VJ, Chaput JP, Janssen I, Katzmarzyk PT, et al Combinations of physical activity, sedentary behaviour and sleep: relationships with health indicators in school-aged children and youth. Appl Physiol Nutr Metab. 2016;41(6 Suppl 3):S283–293. doi: 10.1139/apnm-2015-0626 27306434 10.1139/apnm-2015-0626

[39] Fazah A, Jacob C, Moussa E, El-Hage R, Youssef H, Delamarche P. Activity, inactivity and quality of life among Lebanese adolescents. Pediatr Int. 2010;52(4):573–578. doi: 10.1111/j.1442-200X.2009.03021.x 20030747 10.1111/j.1442-200X.2009.03021.x

[40] Takano M, Matsukura M, Harada K, Wei CN, Ohmori S, Miyakita T, et al Behavior and lifestyle factors related to quality of life in junior high school students. Environ Health Prev Med. 2005;10(2):94–102. doi: 10.1007/BF02897999 21432147 10.1007/BF02897999 PMC2723660

[41] Herman KM, Hopman WM, Sabiston CM. Physical activity, screen time and self-rated health and mental health in Canadian adolescents. Prev Med. 2015;73:112–116. doi: 10.1016/j.ypmed.2015.01.030 25660484 10.1016/j.ypmed.2015.01.030

[42] Strauss RS, Rodzilsky D, Burack G, Colin M. Psychosocial correlates of physical activity in healthy children. Arch Pediatr Adolesc Med. 2001;155(8):897–902. 11483116 10.1001/archpedi.155.8.897

[43] Sharma B, Nam EW, Kim D, Yoon YM, Kim Y, Kim HY. Role of gender, family, lifestyle and psychological factors in self-rated health among urban adolescents in Peru: a school-based cross-sectional survey. BMJ Open. 2016;6(2):e010149 doi: 10.1136/bmjopen-2015-010149 26842274 10.1136/bmjopen-2015-010149 PMC4746445

[44] Tymms PB, Curtis SE, Routen AC, Thomson KH, Bolden DS, Bock S, et al Clustered randomised controlled trial of two education interventions designed to increase physical activity and well-being of secondary school students: the MOVE Project. BMJ Open. 2016;6(1):e009318 doi: 10.1136/bmjopen-2015-009318 26739729 10.1136/bmjopen-2015-009318 PMC4716156

[45] Breslin G, Gossrau-Breen D, McCay N, Gilmore G, McDonald L, Hanna D. Physical activity, gender, weight status, and wellbeing in 9- to 11-year-old children: a cross sectional survey. J Phys Act Health. 2012;9(3):394–401. 21934158 10.1123/jpah.9.3.394

[46] Muros JJ, Salvador Pérez F, Zurita Ortega F, Gámez Sánchez VM, Knox E. The association between healthy lifestyle behaviors and health-related quality of life among adolescents. J Pediatr (Rio J). 2017;93(4):406–412. 28130968 10.1016/j.jped.2016.10.005

[47] Jalali-Farahani S, Amiri P, Chin YS. Are physical activity, sedentary behaviors and sleep duration associated with body mass index-for-age and health-related quality of life among high school boys and girls? Health Qual Life Outcomes. 2016;14:30 doi: 10.1186/s12955-016-0434-6 26921272 10.1186/s12955-016-0434-6 PMC4769527

[48] Omorou AY, Langlois J, Lecomte E, Briançon S, Vuillemin A. Cumulative and bidirectional association of physical activity and sedentary behaviour with health-related quality of life in adolescents. Qual Life Res. 2016;25(5):1169–1178. doi: 10.1007/s11136-015-1172-7 26542533 10.1007/s11136-015-1172-7

[49] Wafa SW, Shahril MR, Ahmad AB, Zainuddin LR, Ismail KF, Aung MM, et al Association between physical activity and health-related quality of life in children: a cross-sectional study. Health Qual Life Outcomes. 2016;14:71 doi: 10.1186/s12955-016-0474-y 27146199 10.1186/s12955-016-0474-y PMC4857334

[50] Sigvartsen J, Gabrielsen LE, Abildsnes E, Stea TH, Omfjord CS, Rohde G. Exploring the relationship between physical activity, life goals and health-related quality of life among high school students: a cross-sectional study. BMC Public Health. 2016;15:709 doi: 10.1186/s12889-016-3407-0 27488255 10.1186/s12889-016-3407-0 PMC4972944

[51] Casey MM, Harvey JT, Telford A, Eime RM, Mooney A, Payne WR. Effectiveness of a school-community linked program on physical activity levels and health-related quality of life for adolescent girls. BMC Public Health. 2014;14:649 doi: 10.1186/1471-2458-14-649 24966134 10.1186/1471-2458-14-649 PMC4080584

[52] Chen G, Ratcliffe J, Olds T, Magarey A, Jones M, Leslie E. BMI, health behaviors, and quality of life in children and adolescents: a school-based study. Pediatrics. 2014;133(4):e868–874. doi: 10.1542/peds.2013-0622 24590749 10.1542/peds.2013-0622

[53] Gopinath B, Louie JC, Flood VM, Burlutsky G, Hardy LL, Baur LA, et al Influence of obesogenic behaviors on health-related quality of life in adolescents. Asia Pac J Clin Nutr. 2014;23(1):121–127. doi: 10.6133/apjcn.2014.23.1.13 24561980 10.6133/apjcn.2014.23.1.13

[54] Vella SA, Cliff DP, Magee CA, Okely AD. Sports participation and parent-reported health-related quality of life in children: longitudinal associations. J Pediatr. 2014;164(6):1469–1474. doi: 10.1016/j.jpeds.2014.01.071 24657117 10.1016/j.jpeds.2014.01.071

[55] Xu F, Chen G, Stevens K, Zhou H, Qi S, Wang Z, et al Measuring and valuing health-related quality of life among children and adolescents in mainland China—a pilot study. PLoS One. 2014; 9(2):e89222 doi: 10.1371/journal.pone.0089222 24586607 10.1371/journal.pone.0089222 PMC3930638

[56] Gu X, Solmon MA, Zhang T. Understanding middle school students’ physical activity and health-related quality of life: An Expectancy-Value Perspective. Applied Research in Quality of Life. 2014;9(4):1041–1054.

[57] Finne E, Bucksch J, Lampert T, Kolip P. Physical activity and screen-based media use: cross-sectional associations with health-related quality of life and the role of body satisfaction in a representative sample of German adolescents. Health Psychol Behav Med. 2013;1(1):15–30. doi: 10.1080/21642850.2013.809313 25264498 10.1080/21642850.2013.809313 PMC4164240

[58] Galán I, Boix R, Medrano MJ, Ramos P, Rivera F, Pastor-Barriuso R, et al Physical activity and self-reported health status among adolescents: a cross-sectional population-based study. BMJ Open. 2013;3:e002644 doi: 10.1136/bmjopen-2013-002644 23676798 10.1136/bmjopen-2013-002644 PMC3657658

[59] Petracci E, Cavrini G. The effect of weight status, lifestyle, and body image perception on health-related quality of life in children: a quantile approach. Qual Life Res. 2013;22(9):2607–2615. doi: 10.1007/s11136-013-0358-0 23423756 10.1007/s11136-013-0358-0

[60] Spengler S, Woll A. The more physically active, the healthier? The relationship between physical activity and health-related quality of life in adolescents: the MoMo study. J Phys Act Health. 2013;10(5):708–715. 23006665 10.1123/jpah.10.5.708

[61] Gopinath B, Hardy LL, Baur LA, Burlutsky G, Mitchell P. Physical activity and sedentary behaviors and health-related quality of life in adolescents. Pediatrics. 2012;130(1):e167–174. doi: 10.1542/peds.2011-3637 22689863 10.1542/peds.2011-3637

[62] Wu XY, Ohinmaa A, Veugelers PJ. Diet quality, physical activity, body weight and health-related quality of life among grade 5 students in Canada. Public Health Nutr. 2012;15(1):75–81. doi: 10.1017/S1368980011002412 22014537 10.1017/S1368980011002412

[63] Perry TT., Moore PC., Redwine KM., Robbins JM., Weber JL. Physical activity, screen time and pediatric health related quality of life in the Mississippi Delta. J of Prev Med. 2012;2(1):1–7.

[64] Lacy KE, Allender SE, Kremer PJ, de Silva-Sanigorski AM, Millar LM, Moodie ML, et al Screen time and physical activity behaviours are associated with health-related quality of life in Australian adolescents. Qual Life Res. 2012;21(6):1085–1099. doi: 10.1007/s11136-011-0014-5 21932139 10.1007/s11136-011-0014-5

[65] Borras PA, Vidal J, Ponseti X, Cantallops J, Palou P. Predictors of quality of life in children. J Hum Sport Exerc. 2011;6(4):649–656.

[66] Dalton WT 3rd, Schetzina KE, Pfortmiller DT, Slawson DL, Frye WS. Health behaviors and health-related quality of life among middle school children in Southern Appalachia: data from the winning with wellness project. J Pediatr Psychol. 2011;36(6):677–686. doi: 10.1093/jpepsy/jsq108 21131337 10.1093/jpepsy/jsq108

[67] Hartmann T, Zahner L, Pühse U, Puder JJ, Kriemler S. Effects of a school-based physical activity program on physical and psychosocial quality of life in elementary school children: a cluster-randomized trial. Pediatr Exerc Sci. 2010;22(4):511–522. 21242601 10.1123/pes.22.4.511

[68] Kriemler S, Zahner L, Schindler C, Meyer U, Hartmann T, Hebestreit H, et al Effect of school based physical activity programme (KISS) on fitness and adiposity in primary schoolchildren: cluster randomised controlled trial. BMJ. 2010;340:c785 doi: 10.1136/bmj.c785 20179126 10.1136/bmj.c785 PMC2827713

[69] Boyle SE, Jones GL, Walters SJ. Physical activity, quality of life, weight status and diet in adolescents. Qual Life Res. 2010;19:943–954. doi: 10.1007/s11136-010-9659-8 20454863 10.1007/s11136-010-9659-8

[70] Gordia AP, Silva RCR, Quadros TMB, Campos Wagner de. Behavioral and sociodemographic variables are associated with the psychological domain of adolescents’ quality of life. Rev Paul Pediatr. 2010;28(1):29–35.

[71] Herman KM, Hopman WM, Craig CL. Are youth BMI and physical activity associated with better or worse than expected health-related quality of life in adulthood? The Physical Activity Longitudinal Study. Qual Life Res. 2010;19(3):339–349. doi: 10.1007/s11136-010-9586-8 20077141 10.1007/s11136-010-9586-8

[72] Mathers M., Canterford L, Olds T, Hesketh K, Ridley K, Wake M. Electronic media use and adolescent health and well-being: Cross-sectional community study. Academic Pediatrics. 2009; 9(5):307–314. doi: 10.1016/j.acap.2009.04.003 19592322 10.1016/j.acap.2009.04.003

[73] Sánchez-López M, Salcedo-Aguilar F, Solera-Martínez M, Moya-Martínez P, Notario-Pacheco B, Martínez-Vizcaíno V. Physical activity and quality of life in schoolchildren aged 11–13 years of Cuenca, Spain. Scand J Med Sci Sports. 2009;19:879–884. doi: 10.1111/j.1600-0838.2008.00839.x 18980609 10.1111/j.1600-0838.2008.00839.x

[74] Wang H, Sekine M, Chen X, Yamagami T, Kagamimori S. Lifestyle at 3 years of age and quality of life (QOL) in first-year junior high school students in Japan: results of the Toyama Birth Cohort Study. Qual Life Res. 2008;17(2):257–265. doi: 10.1007/s11136-007-9301-6 18157615 10.1007/s11136-007-9301-6

[75] Chen X, Sekine M, Hamanishi S, Yamagami T, Kagamimori S. Associations of lifestyle factors with quality of life (QOL) in Japanese children: a 3-year follow-up of the Toyama Birth Cohort Study. Child Care Health Dev. 2005a;31(4):433–439. 15948880 10.1111/j.1365-2214.2005.00529.x

[76] Chen X, Sekine M, Hamanishi S, Wang H, Gaina A, Yamagami T, et al Lifestyles and health-related quality of life in Japanese school children: across-sectional study. Prev Med. 2005b;40(6):668–678. 15850863 10.1016/j.ypmed.2004.09.034

[77] Wille N, Badia X, Bonsel G, Burström K, Cavrini G, Devlin N, et al Development of the EQ-5D-Y: A child-friendly version of the EQ-5D. Qual Life Res. 2010;19(6):875–886. doi: 10.1007/s11136-010-9648-y 20405245 10.1007/s11136-010-9648-y PMC2892611

[78] Ravens-Sieberer U, Wille N, Badia X, Bonsel G, Burström K, Cavrini G, et al Feasibility, reliability, and validity of the EQ-5D-Y: results from a multinational study. Qual Life Res. 2010;19(6):887–897. doi: 10.1007/s11136-010-9649-x 20401552 10.1007/s11136-010-9649-x PMC2892614

[79] Brown DW, Brown DR, Heath GW, Balluz L, Giles WH, Ford ES, et al Associations between physical activity dose and health-related quality of life. Med Sci Sports Exerc. 2004;36(5):890–896. 15126726 10.1249/01.mss.0000126778.77049.76

[80] Jaeschke R, Singer J & Guyatt GH. Measurement of health status. Ascertaining the minimal clinically important difference. Control Clin Trials. 1989;10:407–415. 2691207 10.1016/0197-2456(89)90005-6

[81] Cohen J. Statistical power analysis for the behavioural sciences. 2nd ed Hillsdale: NJ: Laurence Erlbaum; 1988.

[82] Varni JW, Burwinkle TM, Seid M, Skarr D. The PedsQL 4.0 as a pediatric population health meaure: feasibility, reliability, and validity. Ambul Pediatr. 2003;3:329–341. 14616041 10.1367/1539-4409(2003)003<0329:tpaapp>2.0.co;2

[83] Hardy LL, Hills AP, Timperio A, Cliff D, Lubans D, Morgan PJ, et al A hitchhiker's guide to assessing sedentary behaviour among young people: deciding what method to use. J Sci Med Sport. 2013;16(1):28–35. doi: 10.1016/j.jsams.2012.05.010 22749939 10.1016/j.jsams.2012.05.010

[84] Chinapaw MJ, Proper KI, Brug J, van Mechelen W, Singh AS. Relationship between young peoples’ sedentary behaviour and biomedical health indicators: a systematic review of prospective studies. Obes Rev. 2011;12(7):e621–e632. doi: 10.1111/j.1467-789X.2011.00865.x 21438990 10.1111/j.1467-789X.2011.00865.x

[85] School Health Guidelines to Promote Healthy Eating and Physical Activity. Centers for Disease Control and Prevention. US Department of Health and Human Services. 2011. https://www.cdc.gov/healthyschools/npao/pdf/mmwr-school-health-guidelines.pdf. Accessed on May 21,2017.

[86] Tremblay MS, Leblanc AG, Janssen I, Kho ME, Hicks A, Murumets K, et al Canadian sedentary behaviour guidelines for children and youth. Appl Physiol Nutr Metab. 2011;36(1):59–64; 65–71. doi: 10.1139/H11-012 21326378 10.1139/H11-012

[87] Tremblay MS, Warburton DE, Janssen I, Paterson DH, Latimer AE, Rhodes RE, et al New Canadian physical activity guidelines. Appl Physiol Nutr Metab. 2011;36(1):36–46; 47–58. doi: 10.1139/H11-009 21326376 10.1139/H11-009

[88] Global recommendations on physical activity for health. WHO,2010 http://apps.who.int/iris/bitstream/10665/44399/1/9789241599979_eng.pdf. Accessed on May 21,2017. 26180873

[89] Troiano RP, Berrigan D, Dodd KW, Masse LC, Tilert T, McDowell M. Physical activity in the United States measured by accelerometer. Medicine and Science in Sports and Exercise. 2008;40(1):181–188. doi: 10.1249/mss.0b013e31815a51b3 18091006 10.1249/mss.0b013e31815a51b3

[90] McLure SA, Summerbell CD, Reilly JJ. Objectively measured habitual physical activity in a highly obesogenic environment. Child Care Health Development. 2009; 35(3):369–375. 10.1111/j.1365-2214.2009.00946.x 19397599

[91] Wafa SW, Hamzaid H, Talib RA, Reilly JJ. Objectively measured habitual physical activity and sedentary behaviour in obese and non-obese Malaysian children. J Trop Pediatr. 2014;60(2):161–163. doi: 10.1093/tropej/fmt093 24213306 10.1093/tropej/fmt093

[92] Lee S, Wong J, Shanita S, Ismail M, Deurenberg P, Poh B. Daily physical activity and screen time, but not other sedentary activities, are associated with measures of obesity during childhood. Int J Environ Res Public Health. 2014;12(1):146–161. doi: 10.3390/ijerph120100146 25546277 10.3390/ijerph120100146 PMC4306854

[93] Colley RC, Garriguet D, Janssen I, Craig C, Clarke J, Tremblay MS. Physical activity of Canadian children and youth: accelerometer results from the 2007 to 2009 Canadian Health Measures Survey. Health Rep. 2011;22(1):15–23. 21510586

