# Supplementary material for: The influence of physical activity, sedentary behavior on health-related quality of life among the general population of children and adolescents: A systematic review
Source: PLoS One. 2017 Nov 9;12(11):e0187668. doi: 10.1371/journal.pone.0187668 (PMC5679623; doi:10.1371/journal.pone.0187668)
Supplement: S1 Table — (DOC) [file pone.0187668.s001.doc]

**S1 Tables. Literature search strategies for the databases of MEDLINE, EMBASE and PSYCINFO (Table A-Table C)**

**Table A. MEDLINE**

| **No** | **Searching terms** | **Number of identified citations** |
| --- | --- | --- |
| 1 | physical activity.mp. or exp Motor Activity | 316204 |
| 2 | exp Exercise | 168581 |
| 3 | exp Exercise/ or exp Television/ or exp Computers/or exp Sedentary Lifestyle/ or exp Video Games | 283107 |
| 4 | exp Child | 1897991 |
| 5 | exp Adolescent | 1996762 |
| 6 | exp “Quality of Life” | 164506 |
| 7 | exp Health Status | 151632 |
| 8 | 1 or 2 or 3 | 426971 |
| 9 | 4 or 5 | 2983153 |
| 10 | 6 or 7 | 296318 |
| 11 | 8 and 9 and 10 | 3392 |
| 12 | limit 11 to english language | 3108 |

**Table B. EMBASE**

| **No** | **Searching terms** | **Number of identified citations** |
| --- | --- | --- |
| 1 | exp exercise/ or exp physical activity/ or exp leisure | 623149 |
| 2 | exp lifestyle/ or exp sedentary lifestyle | 118955 |
| 3 | exp television/ or screen time.mp. or exp computer/ or exp television viewing | 189919 |
| 4 | video games.mp. or exp video game | 2248 |
| 5 | computer games.mp. or exp video game | 1401 |
| 6 | child | 1653389 |
| 7 | adolescent | 1415751 |
| 8 | quality of life.mp. or exp "quality of life" | 435489 |
| 9 | QOL.mp | 50172 |
| 10 | 1 or 2 or 3 or 4 or 5 | 886019 |
| 11 | 6 or 7 | 2422641 |
| 12 | 8 or 9 | 439476 |
| 13 | 10 and 11 and 12 | 4640 |
| 14 | limit 13 to (human and english language) | 4165 |
| 15 | limit 14 to article | 2545 |

**Table C. PSYCINFO**

| **No** | **Searching terms** | **Number of identified citations** |
| --- | --- | --- |
| 1 | physical activity.mp. or exp Physical Activity | 40601 |
| 2 | exp Sedentary Behavior/ or exp Exercise/ or exp Leisure Time/ or exp Lifestyle/or exp Health Behavior/ or exp Activity Level | 58721 |
| 3 | exp Screen Time/ or exp Computers/ or exp Television | 20870 |
| 4 | video games.mp. or exp Computer Games | 6098 |
| 5 | child.mp | 230659 |
| 6 | adolescent.mp | 123870 |
| 7 | quality of life.mp. or exp "Quality of Life" | 60074 |
| 8 | health status.mp | 15575 |
| 9 | 1 or 2 or 3 or 4 | 94441 |
| 10 | 5 or 6 | 318233 |
| 11 | 7 or 8 | 73139 |
| 12 | 9 and 10 and 11 | 442 |
| 13 | limit 12 to (human and english language) | 418 |
