# Supplementary material for: The influence of physical activity, sedentary behavior on health-related quality of life among the general population of children and adolescents: A systematic review
Source: PLoS One. 2017 Nov 9;12(11):e0187668. doi: 10.1371/journal.pone.0187668 (PMC5679623; doi:10.1371/journal.pone.0187668)
Supplement: S2 Table — (DOC) [file pone.0187668.s002.doc]

**S2 Table. PRISMA checklist**

| **Section/topic** | | | **No.** | | | **Checklist item** | **Reported on page No.** |
| --- | --- | --- | --- | --- | --- | --- | --- |
| **TITLE** | | | | | | |  |
| Title | | | 1 | | | Identify the report as a systematic review, meta-analysis, or both. | Title page |
| **ABSTRACT** | | | | | | |  |
| Structured summary | | | 2 | | | Provide a structured summary including, as applicable: background; objectives; data sources; study eligibility criteria, participants, and interventions; study appraisal and synthesis methods; results; limitations; conclusions and implications of key findings; systematic review registration number. | Abstract |
| **INTRODUCTION** | | | | | | |  |
| Rationale | | | 3 | | | Describe the rationale for the review in the context of what is already known. | Page 1-2 |
| Objectives | | | 4 | | | Provide an explicit statement of questions being addressed with reference to participants, interventions, comparisons, outcomes, and study design (PICOS). | Page 2  P-Population of children and adolescents;  I or exposure- Physical activity (PA), sedentary behaviour (SB);  C-associations between PA, sedentary behaviour and HRQOL;  O- HRQOL. |
| **METHODS** | | | | | | |  |
| Protocol and registration | 5 | | | | Indicate if a review protocol exists, if and where it can be accessed (e.g., Web address), and, if available, provide registration information including registration number. | | Review proposal was not registered |
| Eligibility criteria | 6 | | | | Specify study characteristics (e.g., PICOS, length of follow-up) and report characteristics (e.g., years considered, language, publication status) used as criteria for eligibility, giving rationale. | | Page 2-3: Inclusion and exclusion criteria |
| Information sources | 7 | | | | Describe all information sources (e.g., databases with dates of coverage, contact with study authors to identify additional studies) in the search and date last searched. | | Page 2: Literature search |
| Search | 8 | | | | Present full electronic search strategy for at least one database, including any limits used, such that it could be repeated. | | Supporting information: S1 Tables for literature search strategy for the electronic databases of Medline, Embase and PsycInfo. |
| Study selection | 9 | | | | State the process for selecting studies (i.e., screening, eligibility, included in systematic review, and, if applicable, included in the meta-analysis). | | Page 2 literature search and Fig 1 PRISMA diagram |
| Data collection process | 10 | | | | Describe method of data extraction from reports (e.g., piloted forms, independently, in duplicate) and any processes for obtaining and confirming data from investigators. | | Page 3-4: Data extraction |
| Data items | 11 | | | | List and define all variables for which data were sought (e.g., PICOS, funding sources) and any assumptions and simplifications made. | | Page 3-4: Data extraction (including PICOS);  Page 4: Data synthesis |
| Risk of bias in individual studies | 12 | | | | Describe methods used for assessing risk of bias of individual studies (including specification of whether this was done at the study or outcome level), and how this information is to be used in any data synthesis. | | Page 4: Risk of bias score |
| Summary measures | 13 | | | | State the principal summary measures (e.g., risk ratio, difference in means). | | Page 4: Data synthesis:  The principal summary measure was the difference in total HRQOL scores (means) between comparison groups by PA and sedentary behaviour. |
| Synthesis of results | 14 | | | | Describe the methods of handling data and combining results of studies, if done, including measures of consistency (e.g., I^2^) for each meta-analysis. | | Page 4: Data synthesis of Meta-analyses |
| Risk of bias across studies | 15 | | | | Specify any assessment of risk of bias that may affect the cumulative evidence (e.g., publication bias, selective reporting within studies). | | Page 4: Risk of bias assessment: grouping of risk of bias scores across-studies |
| Additional analyses | 16 | | | | Describe methods of additional analyses (e.g., sensitivity or subgroup analyses, meta-regression), if done, indicating which were pre-specified. | | Not applicable |
| **RESULTS** | | | | | | |  |
| Study selection | | 17 | | Give numbers of studies screened, assessed for eligibility, and included in the review, with reasons for exclusions at each stage, ideally with a flow diagram. | | | Page 4-5: Characteristics of the included primary studies; and Fig.1: the flow diagram |
| Study characteristics | | 18 | | For each study, present characteristics for which data were extracted (e.g., study size, PICOS, follow-up period) and provide the citations. | | | Page 4-5 and Table 1 |
| Risk of bias within studies | | 19 | | Present data on risk of bias of each study and, if available, any outcome level assessment (see item 12). | | | Page 6-7: Risk of bias assessment, and Table 2: Risk of bias scores |
| Results of individual studies | | 20 | | For all outcomes considered (benefits or harms), present, for each study: (a) simple summary data for each intervention group (b) effect estimates and confidence intervals, ideally with a forest plot. | | | Page 7-11: Results;  Table 1 and Table 2 |
| Synthesis of results | | 21 | | Present results of each meta-analysis done, including confidence intervals and measures of consistency. | | | Page 10-11: Results: Findings from the meta-analysis;  Figure 2-3: Forest plots |
| Risk of bias across studies | | 22 | | Present results of any assessment of risk of bias across studies (see Item 15). | | | Page 6-7: summary results of risk of bias assessment |
| Additional analysis | | 23 | | Give results of additional analyses, if done (e.g., sensitivity or subgroup analyses, meta-regression [see Item 16]). | | | Not applicable |
| **DISCUSSION** | | | | | | |  |
| Summary of evidence | | 24 | | Summarize the main findings including the strength of evidence for each main outcome; consider their relevance to key groups (e.g., healthcare providers, users, and policy makers). | | | Page 11-15 |
| Limitations | | 25 | | Discuss limitations at study and outcome level (e.g., risk of bias), and at review-level (e.g., incomplete retrieval of identified research, reporting bias). | | | Page 14-15: Limitations |
| Conclusions | | 26 | | Provide a general interpretation of the results in the context of other evidence, and implications for future research. | | | Page 15-16: Conclusions |
| **FUNDING** | | | | | | |  |
| Funding | | 27 | | Describe sources of funding for the systematic review and other support (e.g., supply of data); role of funders for the systematic review. | | | Page 18 |
